# Supplementary material for: Dynamics of transposable element accumulation in the non-recombining regions of mating-type chromosomes in anther-smut fungi
Source: Nat Commun. 2023 Sep 14;14:5692. doi: 10.1038/s41467-023-41413-4 (PMC10502011; doi:10.1038/s41467-023-41413-4)
Supplement: Supplementary file 1 — Supplementary Information [file 41467_2023_41413_MOESM1_ESM.pdf]

Supplementary Figures 1-21.

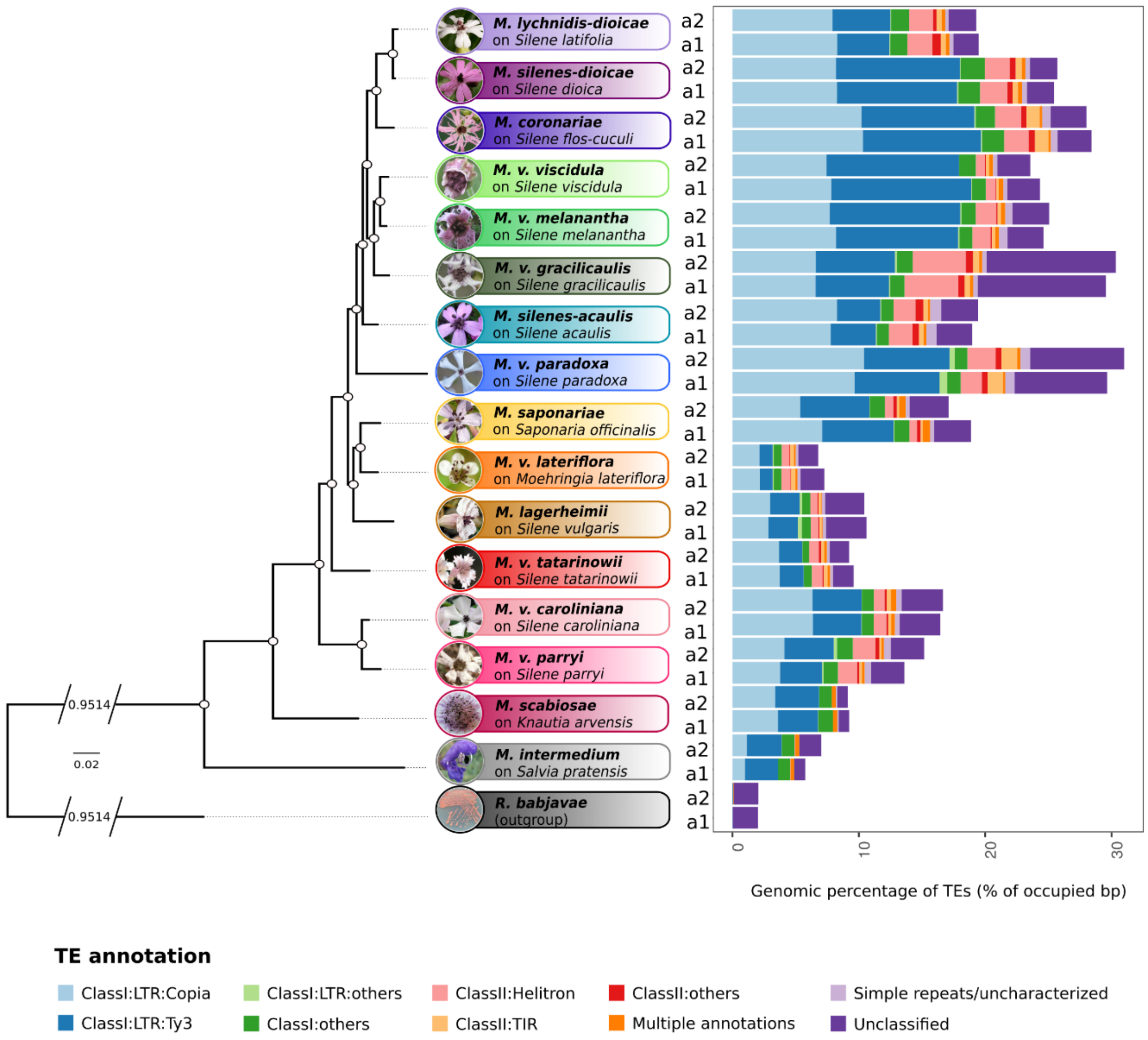

Supplementary Figure 1: Percentage of base pairs occupied by transposable elements (TEs) in *Microbotryum* genomes, both for a1 and a2 mating-type chromosomes. Whole-genome TE content across the *Microbotryum* phylogeny, for each mating-type genome, each color representing a TE category.

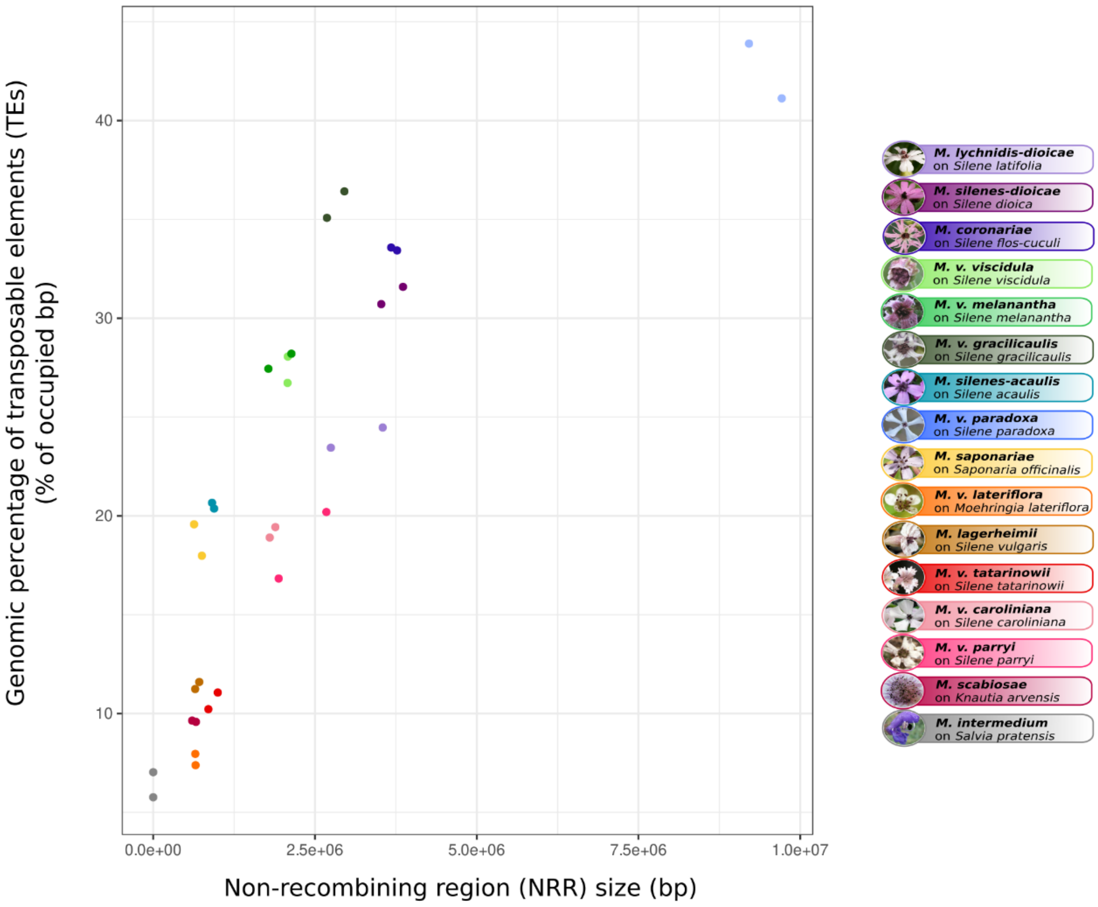

Supplementary Figure 2: Relationship between the percentage of base pairs occupied by TEs and the total size of non-recombining regions. Each color represents one species, one point per mating type.

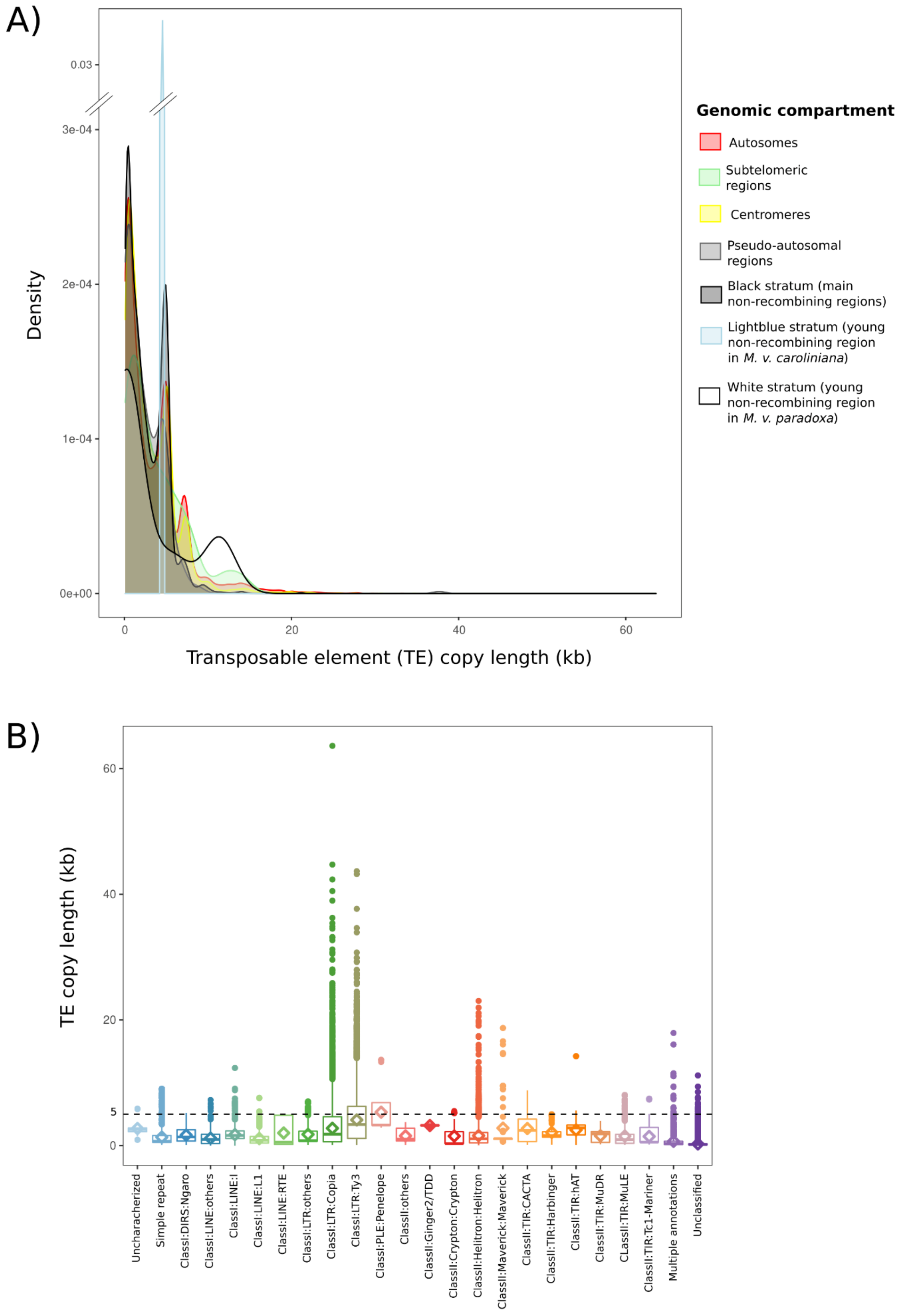
A)
0.03
3e-04
2e-04
1e-04
0e+00
Density
0
20
40
60
Transposable element (TE) copy length (kb)
Genomic compartment
Autosomes
Subtelomeric regions
Centromeres
Pseudo-autosomal regions
Black stratum (main non-recombining regions)
Lightblue stratum (young non-recombining region in M. v. caroliniana)
White stratum (young non-recombining region in M. v. paradoxa)
B)
TE copy length (kb)
60
40
20
5
0
Uncharacherized
Simple repeat
ClassI:DIRS:Ngaro
ClassI:LINE:others
ClassI:LINE:I
ClassI:LINE:L1
ClassI:LINE:RTE
ClassI:LTR:others
ClassI:LTR:Copia
ClassI:LTR:Ty3
ClassI:PLE:Penelope
ClassII:others
ClassII:Ginger2/TDD
ClassII:Crypton:Crypton
ClassII:Helitron:Helitron
ClassII:Maverick:Maverick
ClassII:TIR:CACTA
ClassII:TIR:Harbinger
ClassII:TIR:hAT
ClassII:TIR:MuDR
ClassII:TIR:MuLE
ClassII:TIR:Tc1-Mariner
Multiple annotations
Unclassified
Transposable element (TE) annotation

Supplementary Figure 3: Distribution of transposable element (TE) copy length in *Microbotryum* genomes. A) Distribution of the *Copia* and *Ty3* retrotransposon copy length in the different genomic compartments in various *Microbotryum* species. Note that the Y axis is interrupted by parallel bars. **B)** Distribution of the length of individual TE copies for each TE annotation, across all *Microbotryum* genomes (n = 178,285 independent TE copies). Each color corresponds to a specific annotation. The dashed line indicates the 5 kb expected mean length of *Copia* and *Ty3* retroelements. Similar annotations representing a small proportion of the *Microbotryum* genomes were pooled in all the other figures; e.g., *ClassII:TIR* with two inverted repeats corresponds to all the *TIR* elements, i.e., *CACTA, Harbinger, hAT, MuDR, MuLE* and *Tc1-Mariner*. Boxplots indicate: first quartile Q1 (lower bar of the box), median (line within the box), mean (diamond), (third quartile Q3 (upper bar of the box), Q1-1.5*IQR (lower whisker), Q3+1.5*IQR (upper whisker) and dots are outliers.

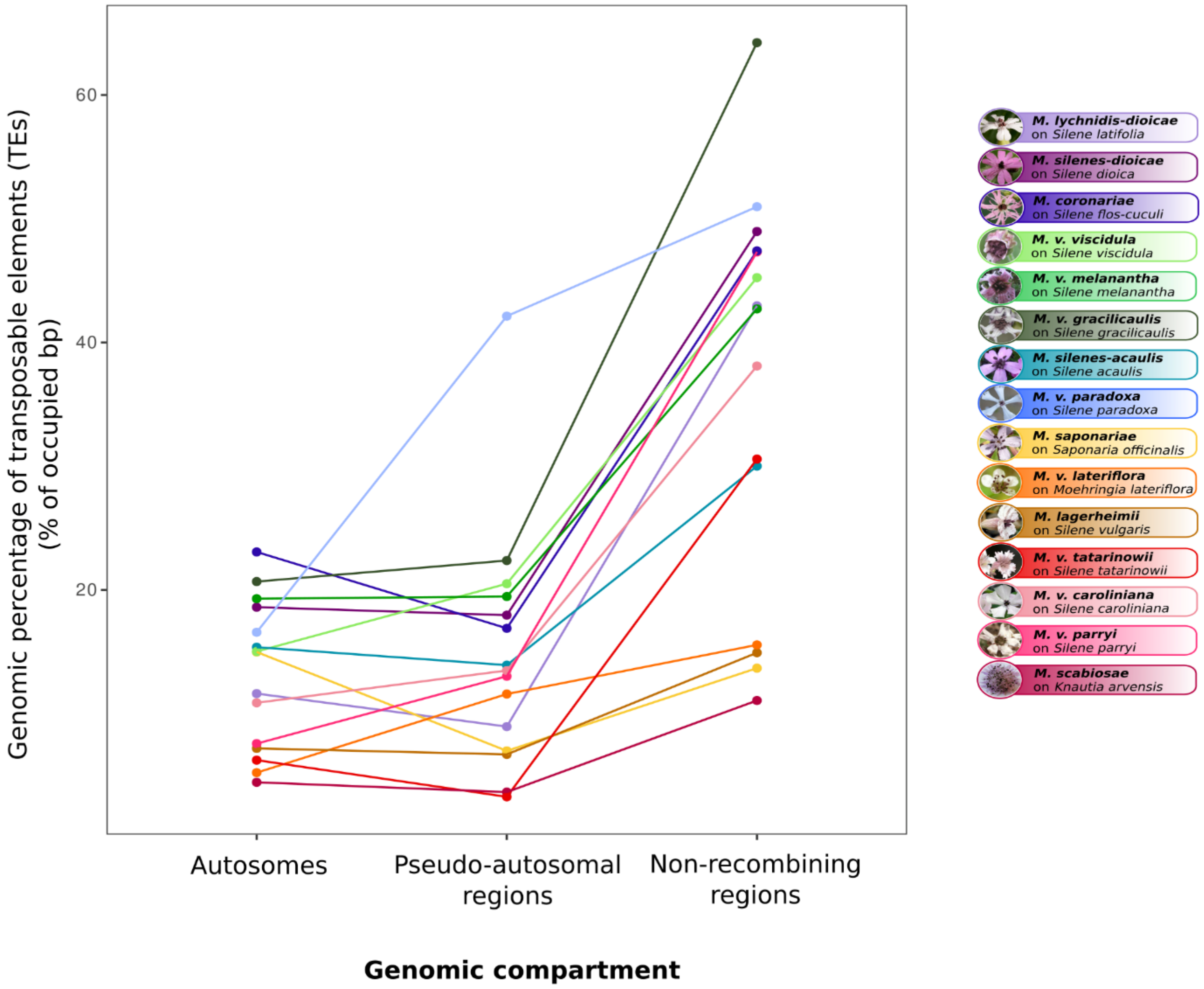

Supplementary Figure 4: Transposable element (TE) proportions in the different genomic compartments of *Microbotryum* fungi. Total TE load per genomic compartment, i.e., autosomes (without the centromeres), pseudo-autosomal regions and non-recombining regions. The lines connect the values in the same species to facilitate comparisons. Each color represents a *Microbotryum* species.

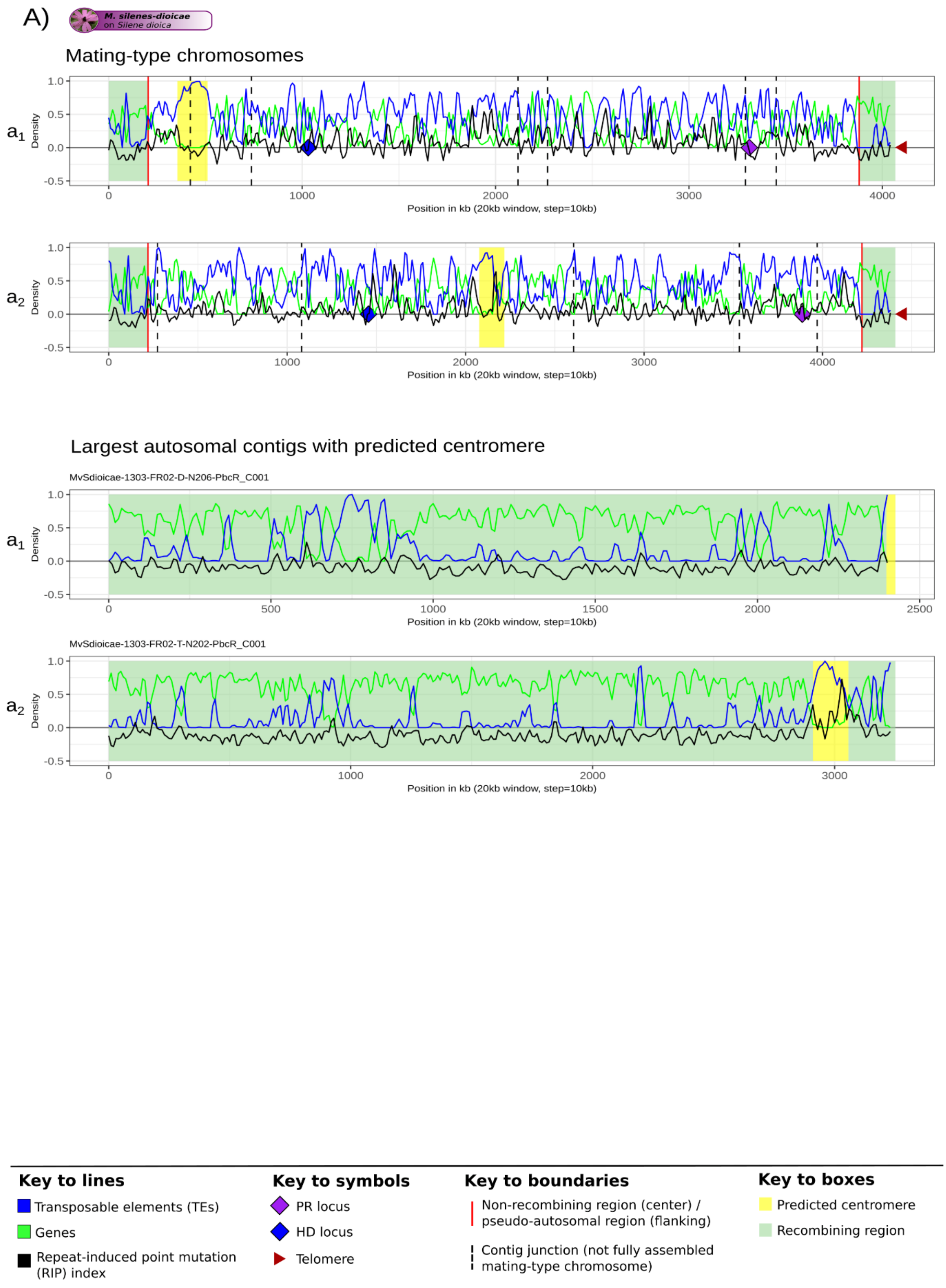
A)
M. silenes-dioicae
on Silene dioica
Mating-type chromosomes
a1
a2
Density
Position in kb (20kb window, step=10kb)
Largest autosomal contigs with predicted centromere
MvSdioicae-1303-FR02-D-N206-PbcR_C001
MvSdioicae-1303-FR02-T-N202-PbcR_C001
Key to lines
Transposable elements (TEs)
Genes
Repeat-induced point mutation (RIP) index
Key to symbols
PR locus
HD locus
Telomere
Key to boundaries
Non-recombining region (center) / pseudo-autosomal region (flanking)
Contig junction (not fully assembled mating-type chromosome)
Key to boxes
Predicted centromere
Recombining region

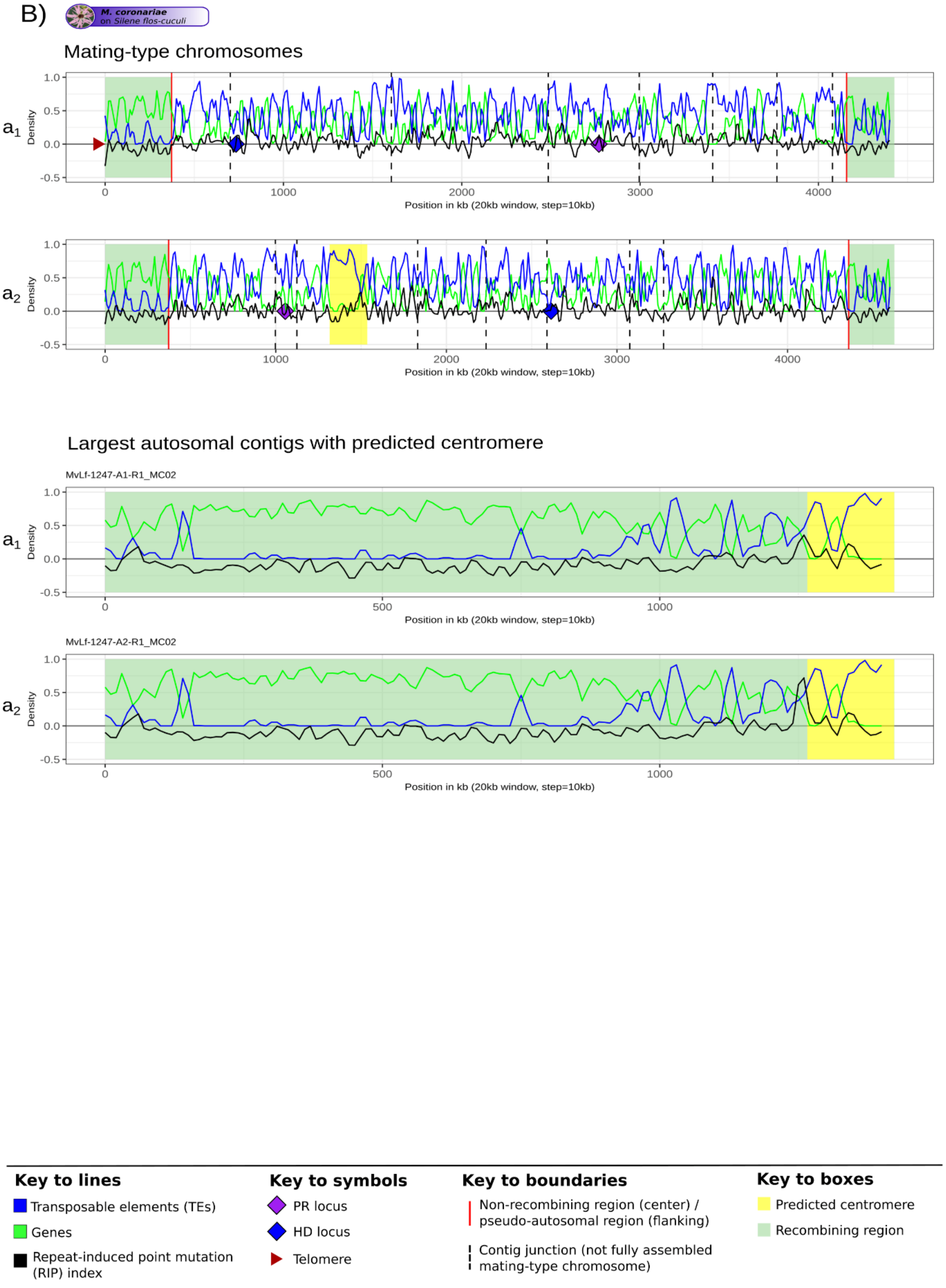
B)
M. coronariae
on Silene flos-cuculi
Mating-type chromosomes
a1
a2
Density
Position in kb (20kb window, step=10kb)
Largest autosomal contigs with predicted centromere
MvLf-1247-A1-R1_MC02
MvLf-1247-A2-R1_MC02
Key to lines
Transposable elements (TEs)
Genes
Repeat-induced point mutation (RIP) index
Key to symbols
PR locus
HD locus
Telomere
Key to boundaries
Non-recombining region (center) / pseudo-autosomal region (flanking)
Contig junction (not fully assembled mating-type chromosome)
Key to boxes
Predicted centromere
Recombining region

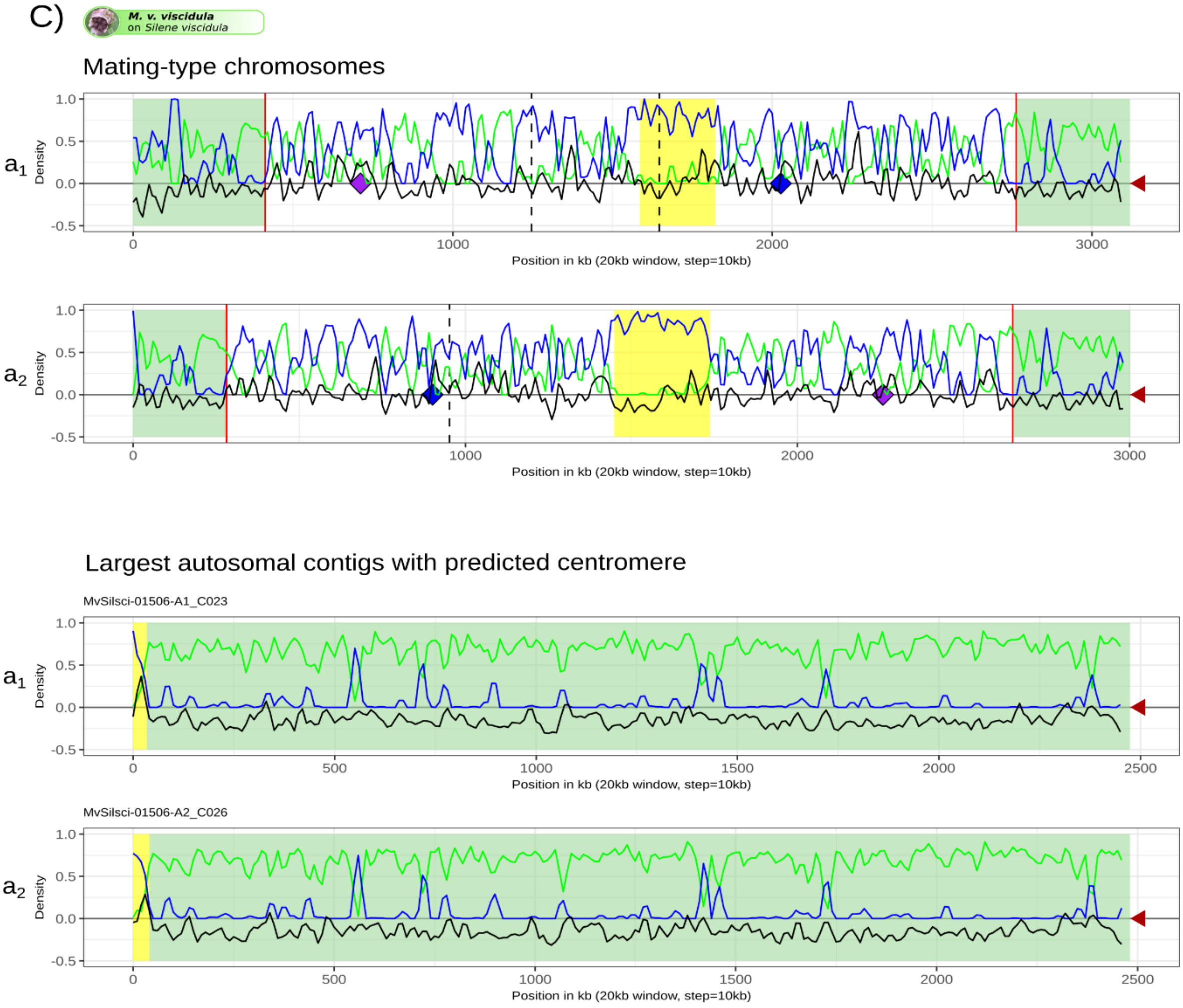

Key to lines

- Transposable elements (TEs)
- Genes
- Repeat-induced point mutation (RIP) index

Key to symbols

- PR locus
- HD locus
- Telomere

Key to boundaries

- Non-recombining region (center) / pseudo-autosomal region (flanking)
- Contig junction (not fully assembled mating-type chromosome)

Key to boxes

- Predicted centromere
- Recombining region

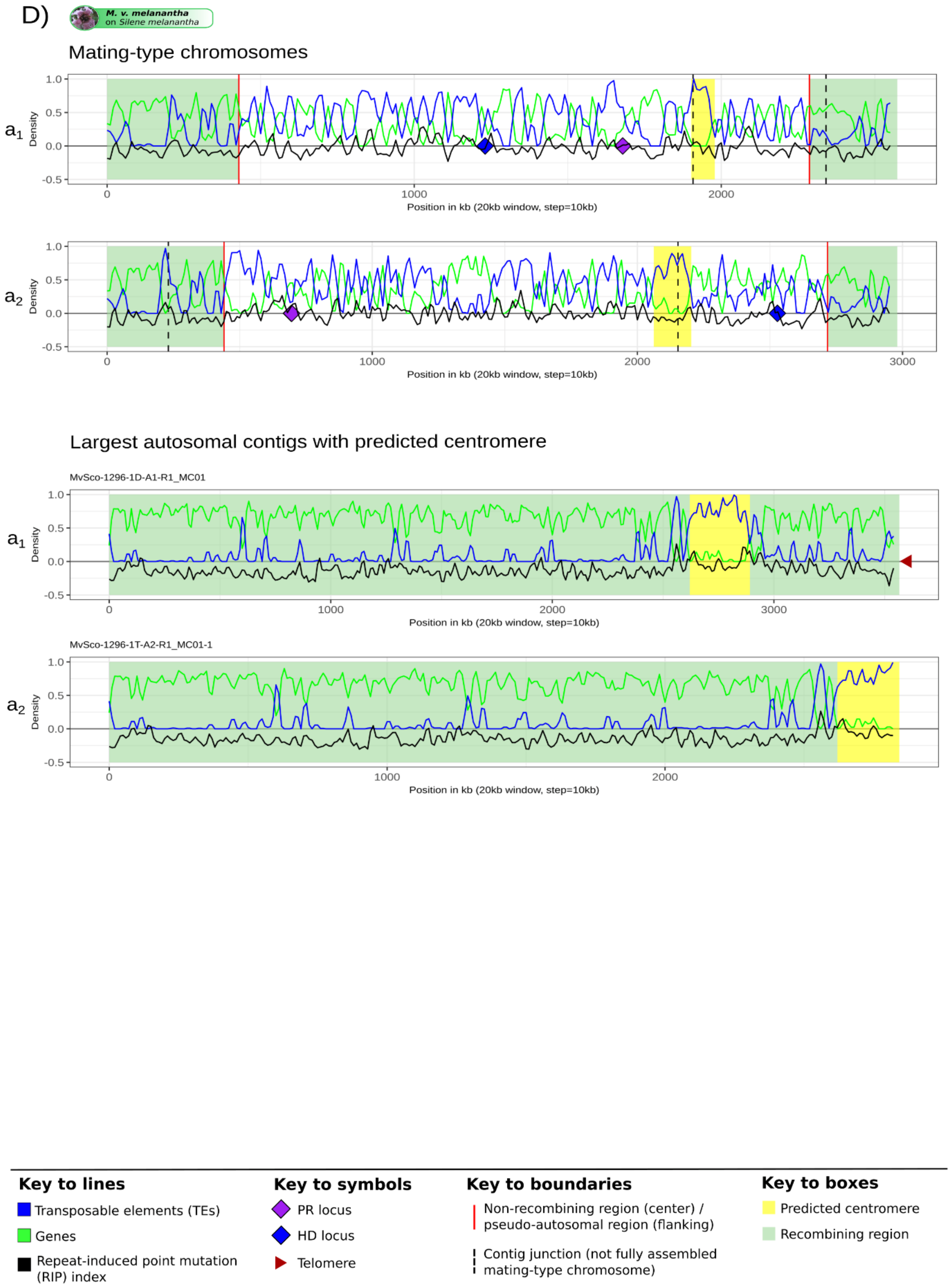
D)
M. v. melanantha
on Silene melanantha
Mating-type chromosomes
a1
a2
Density
1.0
0.5
0.0
-0.5
0
1000
2000
3000
Position in kb (20kb window, step=10kb)
Largest autosomal contigs with predicted centromere
MvSco-1296-1D-A1-R1_MC01
MvSco-1296-1T-A2-R1_MC01-1
Key to lines
Transposable elements (TEs)
Genes
Repeat-induced point mutation (RIP) index
Key to symbols
PR locus
HD locus
Telomere
Key to boundaries
Non-recombining region (center) / pseudo-autosomal region (flanking)
Contig junction (not fully assembled mating-type chromosome)
Key to boxes
Predicted centromere
Recombining region

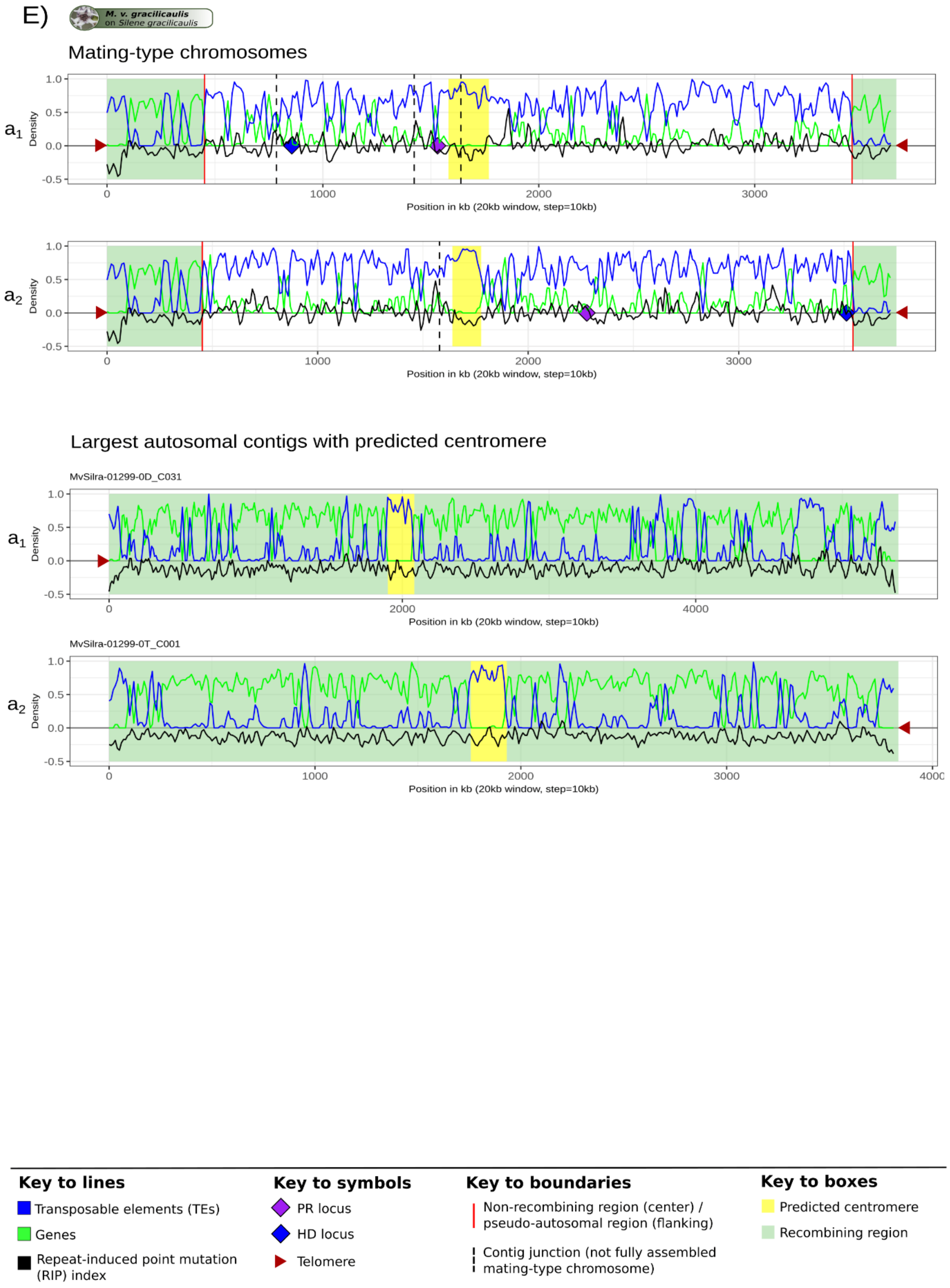
E)
M. v. gracilicaulis
on Silene gracilicaulis
Mating-type chromosomes
a1
a2
Density
1.0
0.5
0.0
-0.5
0
1000
2000
3000
4000
Position in kb (20kb window, step=10kb)
Largest autosomal contigs with predicted centromere
MvSilra-01299-0D_C031
MvSilra-01299-0T_C001
Key to lines
Transposable elements (TEs)
Genes
Repeat-induced point mutation (RIP) index
Key to symbols
PR locus
HD locus
Telomere
Key to boundaries
Non-recombining region (center) / pseudo-autosomal region (flanking)
Contig junction (not fully assembled mating-type chromosome)
Key to boxes
Predicted centromere
Recombining region

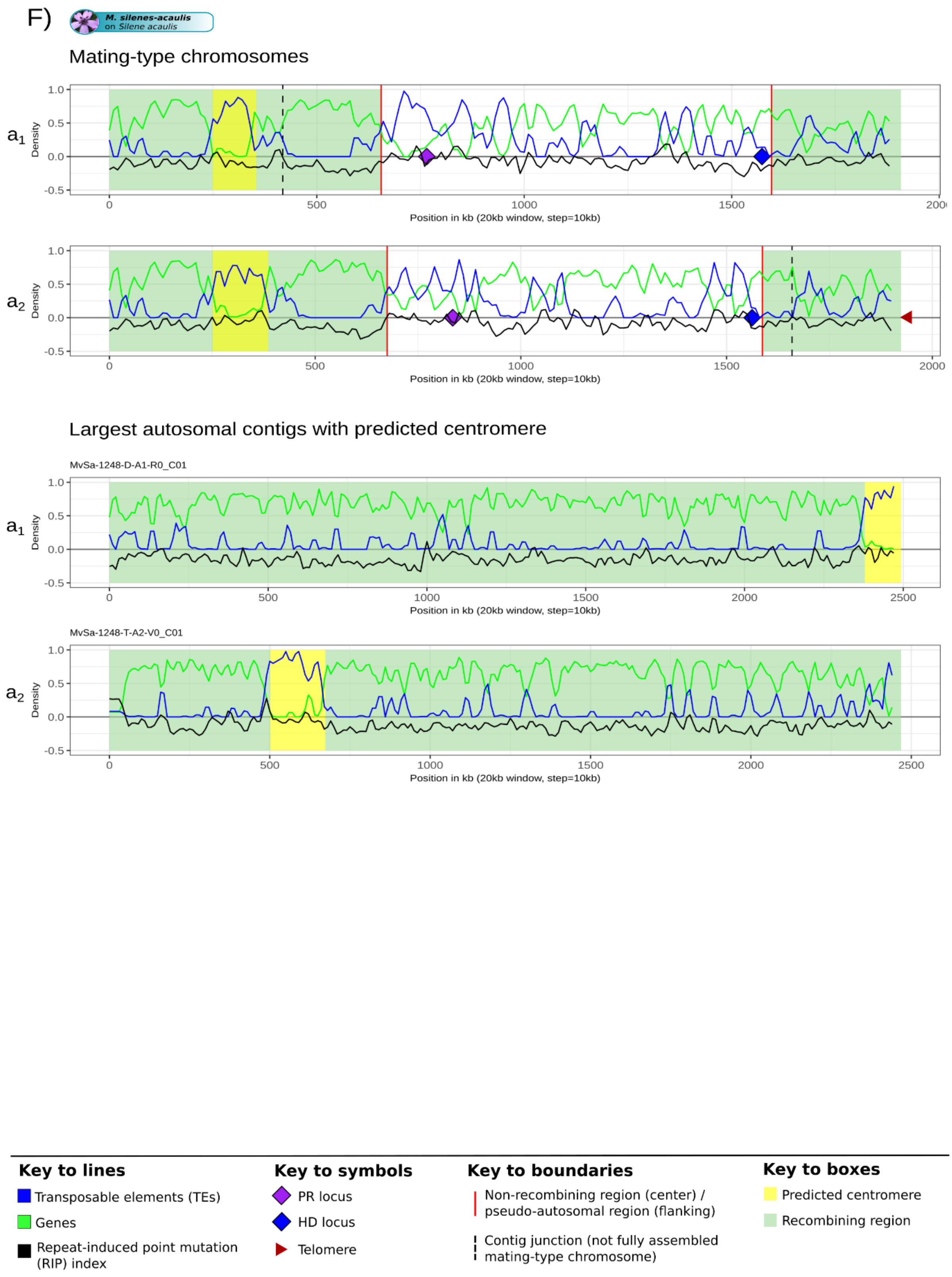
F)
M. silenes-acaulis
on Silene acaulis
Mating-type chromosomes
a1
Density
Position in kb (20kb window, step=10kb)
a2
Largest autosomal contigs with predicted centromere
MvSa-1248-D-A1-R0_C01
MvSa-1248-T-A2-V0_C01
Key to lines
Transposable elements (TEs)
Genes
Repeat-induced point mutation (RIP) index
Key to symbols
PR locus
HD locus
Telomere
Key to boundaries
Non-recombining region (center) / pseudo-autosomal region (flanking)
Contig junction (not fully assembled mating-type chromosome)
Key to boxes
Predicted centromere
Recombining region

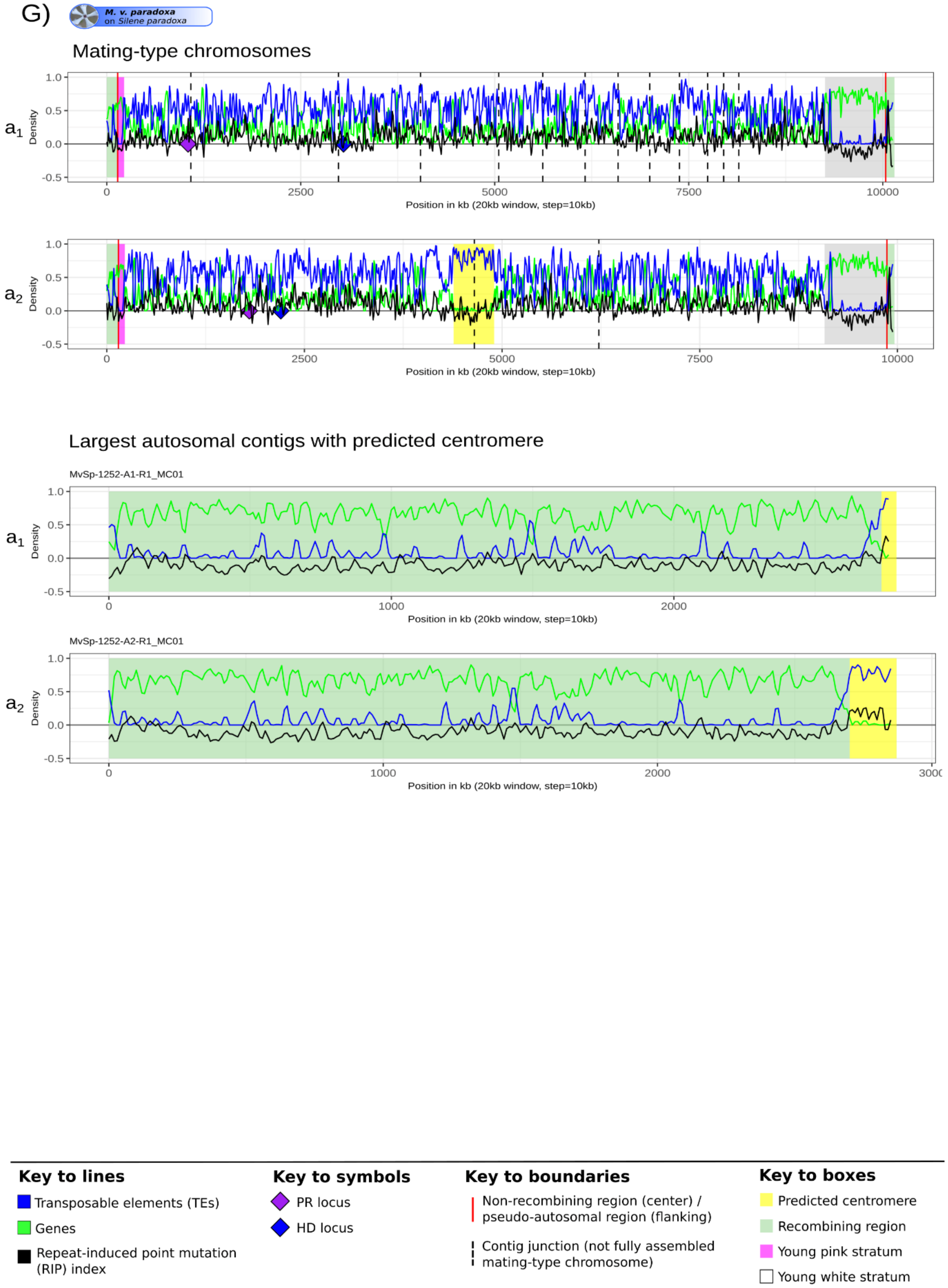
G)
M. v. paradoxa
on Silene paradoxa
Mating-type chromosomes
a1
a2
Density
Position in kb (20kb window, step=10kb)
Largest autosomal contigs with predicted centromere
MvSp-1252-A1-R1_MC01
MvSp-1252-A2-R1_MC01
Key to lines
Transposable elements (TEs)
Genes
Repeat-induced point mutation (RIP) index
Key to symbols
PR locus
HD locus
Key to boundaries
Non-recombining region (center) / pseudo-autosomal region (flanking)
Contig junction (not fully assembled mating-type chromosome)
Key to boxes
Predicted centromere
Recombining region
Young pink stratum
Young white stratum

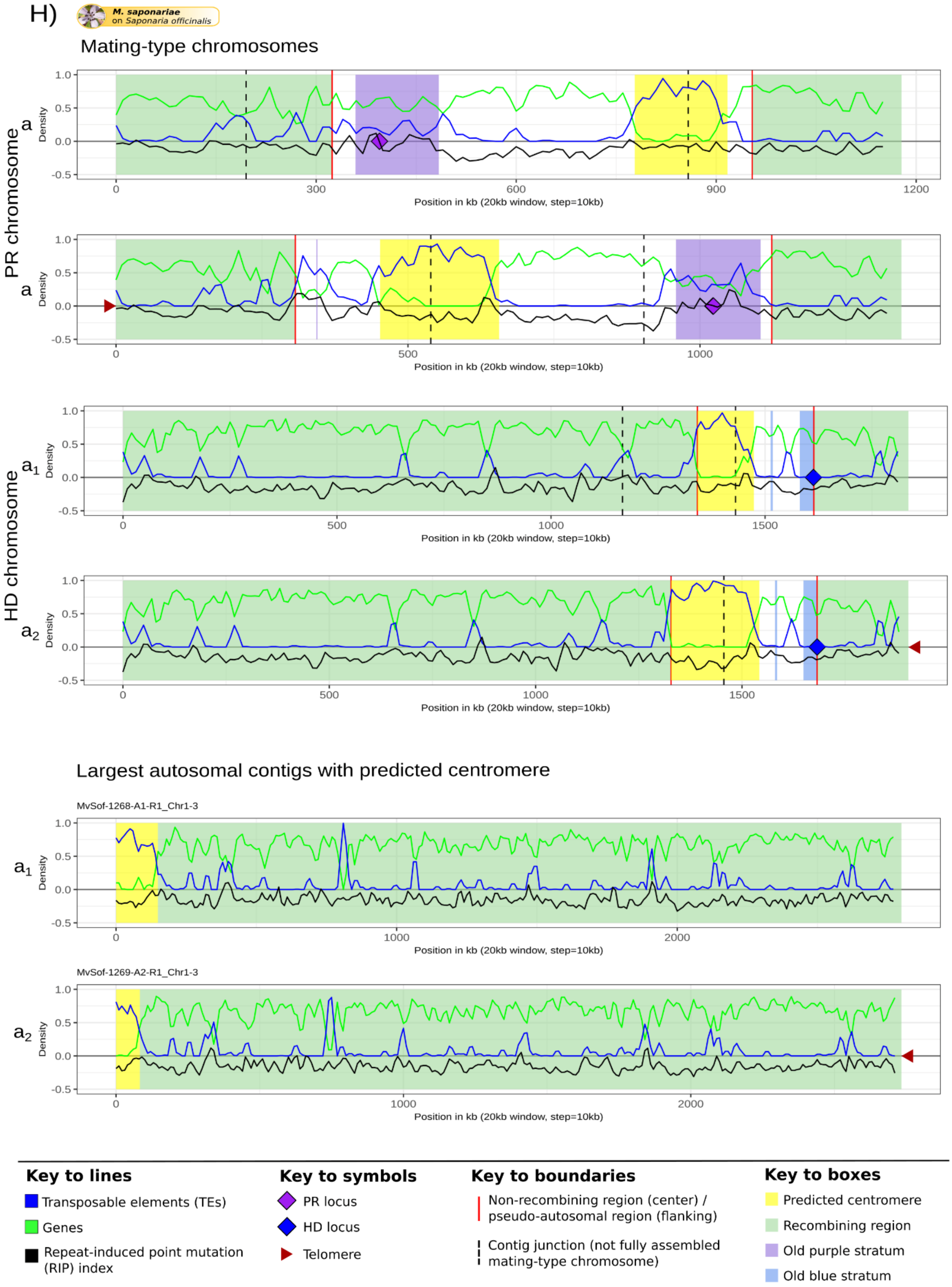

H)
M. saponariae
on Saponaria officinalis
Mating-type chromosomes
PR chromosome
a
a
HD chromosome
a1
a2
Density
Position in kb (20kb window, step=10kb)
Largest autosomal contigs with predicted centromere
MvSof-1268-A1-R1_Chr1-3
MvSof-1269-A2-R1_Chr1-3
Key to lines
Transposable elements (TEs)
Genes
Repeat-induced point mutation (RIP) index
Key to symbols
PR locus
HD locus
Telomere
Key to boundaries
Non-recombining region (center) / pseudo-autosomal region (flanking)
Contig junction (not fully assembled mating-type chromosome)
Key to boxes
Predicted centromere
Recombining region
Old purple stratum
Old blue stratum

I)

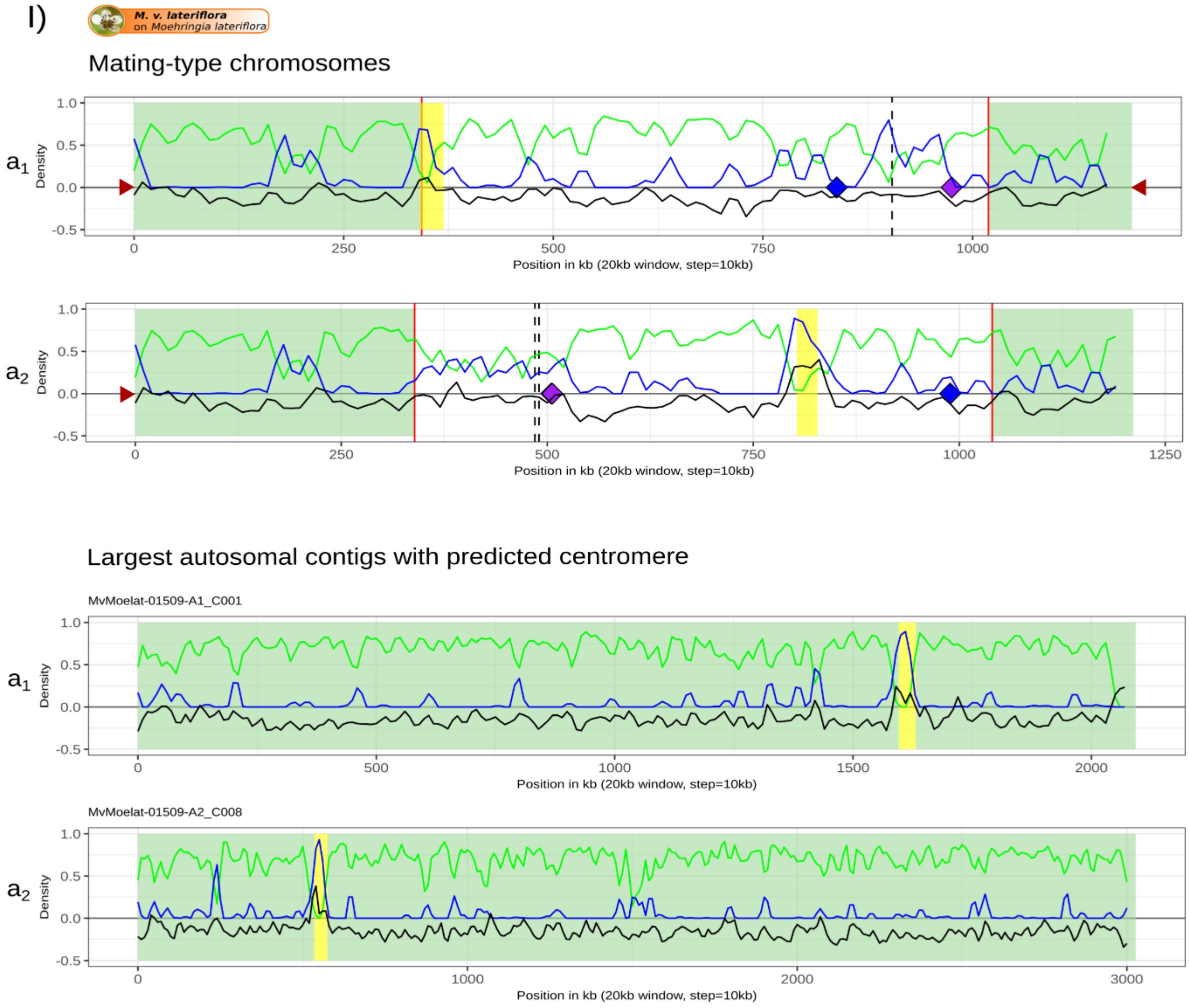

Key to lines

- Transposable elements (TEs)
- Genes
- Repeat-induced point mutation (RIP) index

Key to symbols

- PR locus
- HD locus
- Telomere

Key to boundaries

- Non-recombining region (center) / pseudo-autosomal region (flanking)
- Contig junction (not fully assembled mating-type chromosome)

Key to boxes

- Predicted centromere
- Recombining region
- Old purple stratum
- Old blue stratum

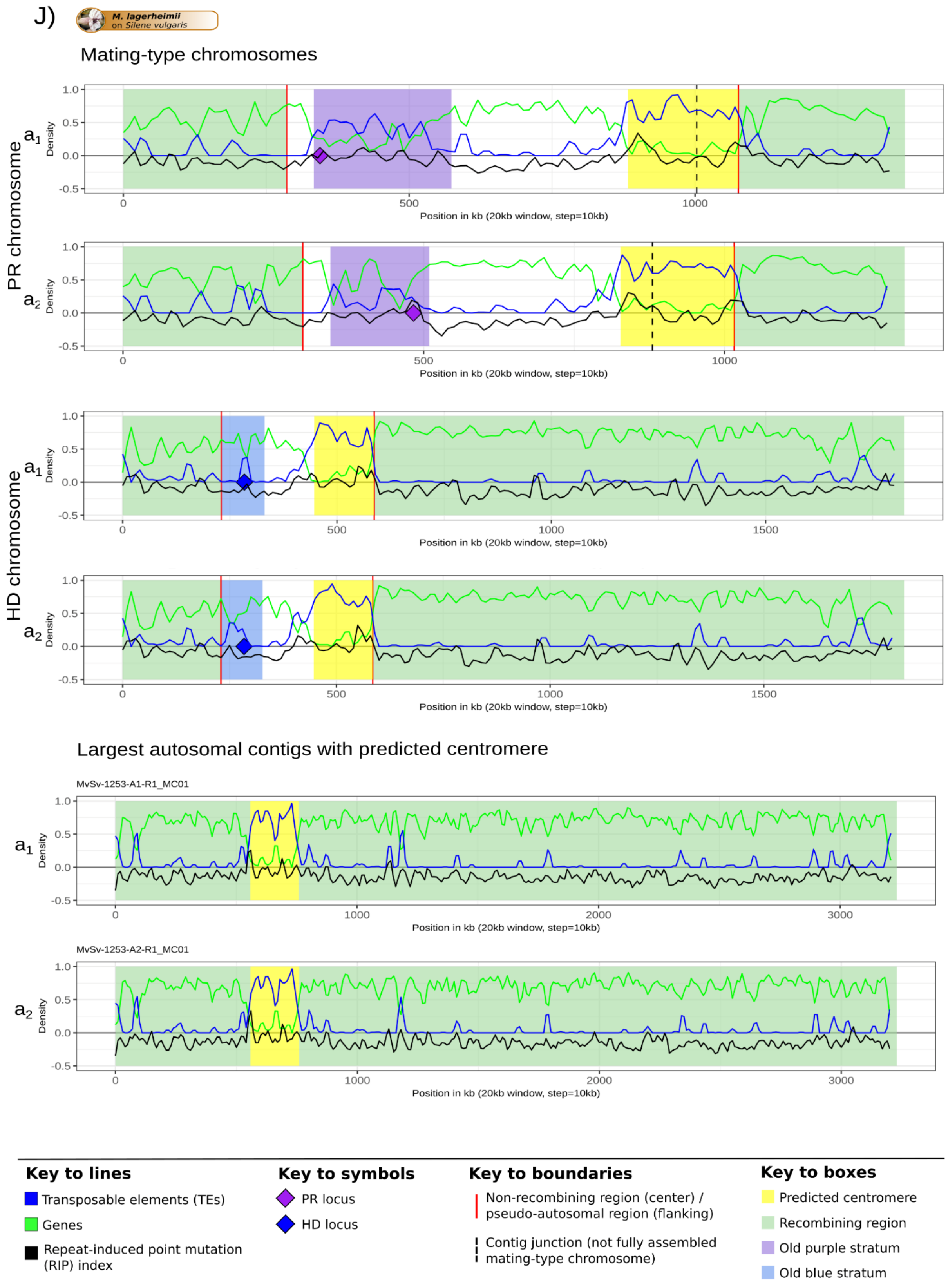
J)
M. lagerheimii on Silene vulgaris
Mating-type chromosomes
PR chromosome
HD chromosome
a1
a2
Density
Position in kb (20kb window, step=10kb)
Largest autosomal contigs with predicted centromere
MvSv-1253-A1-R1_MC01
MvSv-1253-A2-R1_MC01
Key to lines
Transposable elements (TEs)
Genes
Repeat-induced point mutation (RIP) index
Key to symbols
PR locus
HD locus
Key to boundaries
Non-recombining region (center) / pseudo-autosomal region (flanking)
Contig junction (not fully assembled mating-type chromosome)
Key to boxes
Predicted centromere
Recombining region
Old purple stratum
Old blue stratum

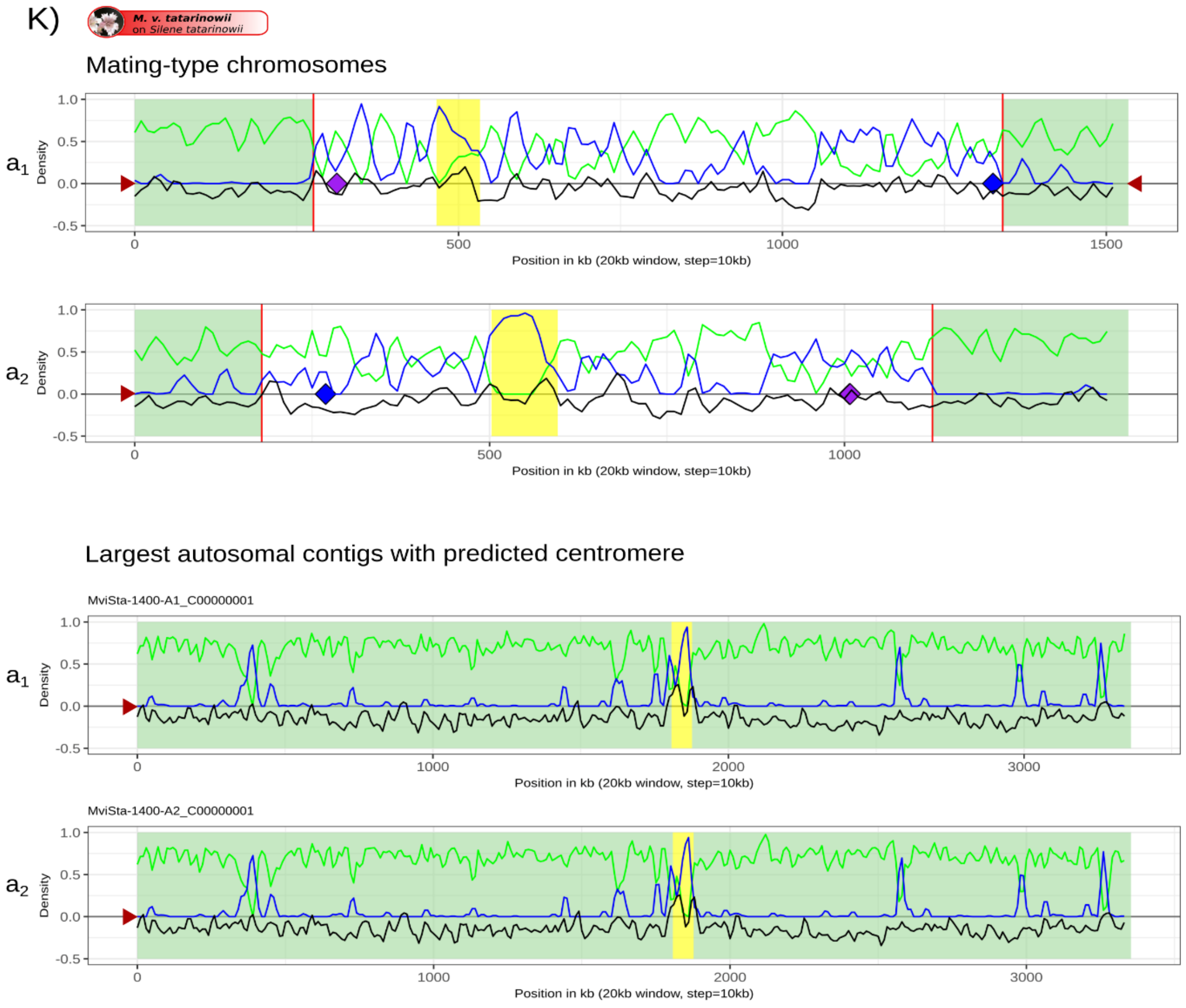

Key to lines
- Transposable elements (TEs)
- Genes
- Repeat-induced point mutation (RIP) index

Key to symbols
- PR locus
- HD locus
- Telomere

Key to boundaries
- Non-recombining region (center) / pseudo-autosomal region (flanking)

Key to boxes
- Predicted centromere
- Recombining region
- Old purple stratum
- Old blue stratum

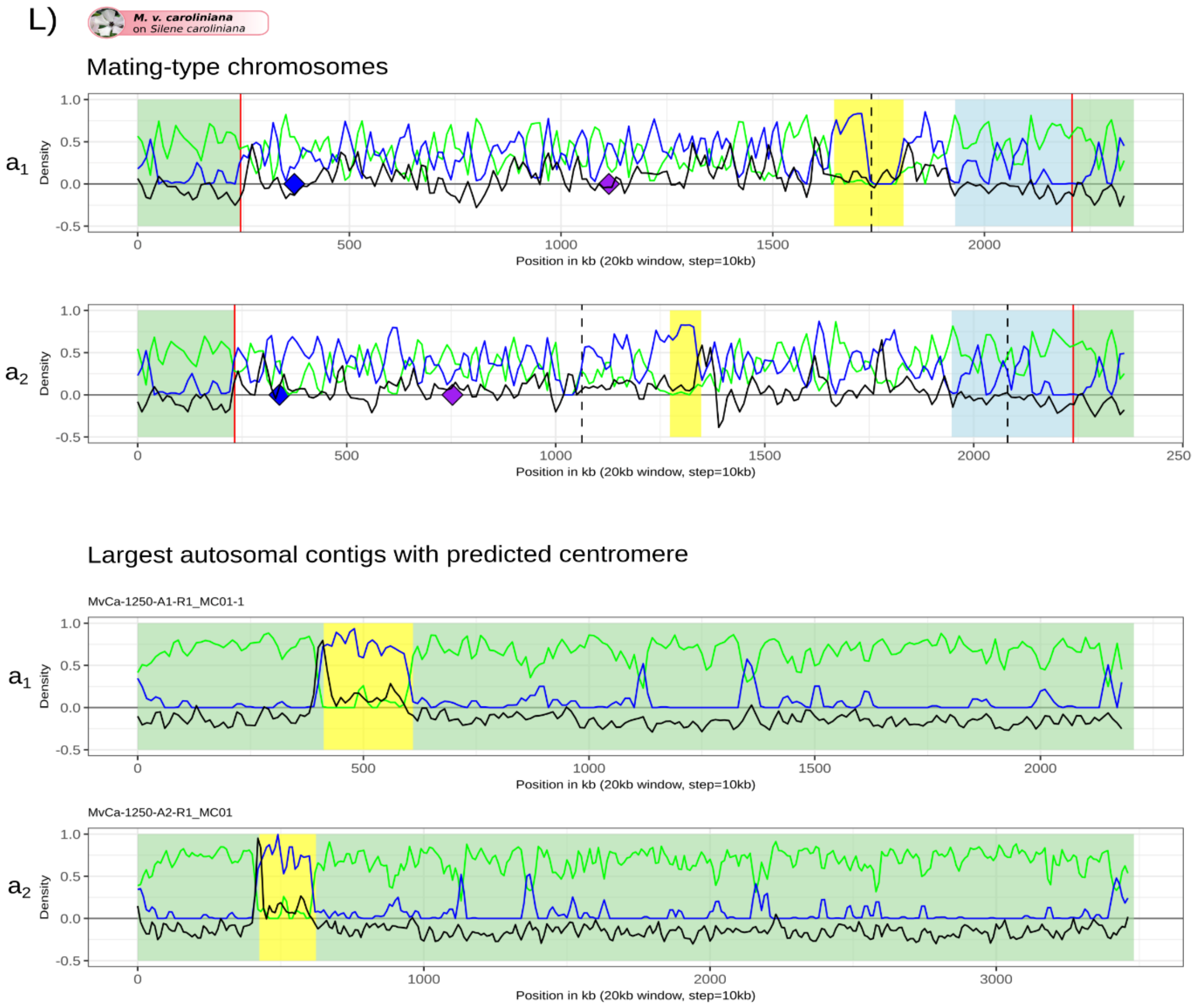

Key to lines

Transposable elements (TEs)

Genes

Repeat-induced point mutation (RIP) index

Key to symbols

PR locus

HD locus

Key to boundaries

Non-recombining region (center) / pseudo-autosomal region (flanking)

Contig junction (not fully assembled mating-type chromosome)

Key to boxes

Predicted centromere

Recombining region

Young light blue stratum

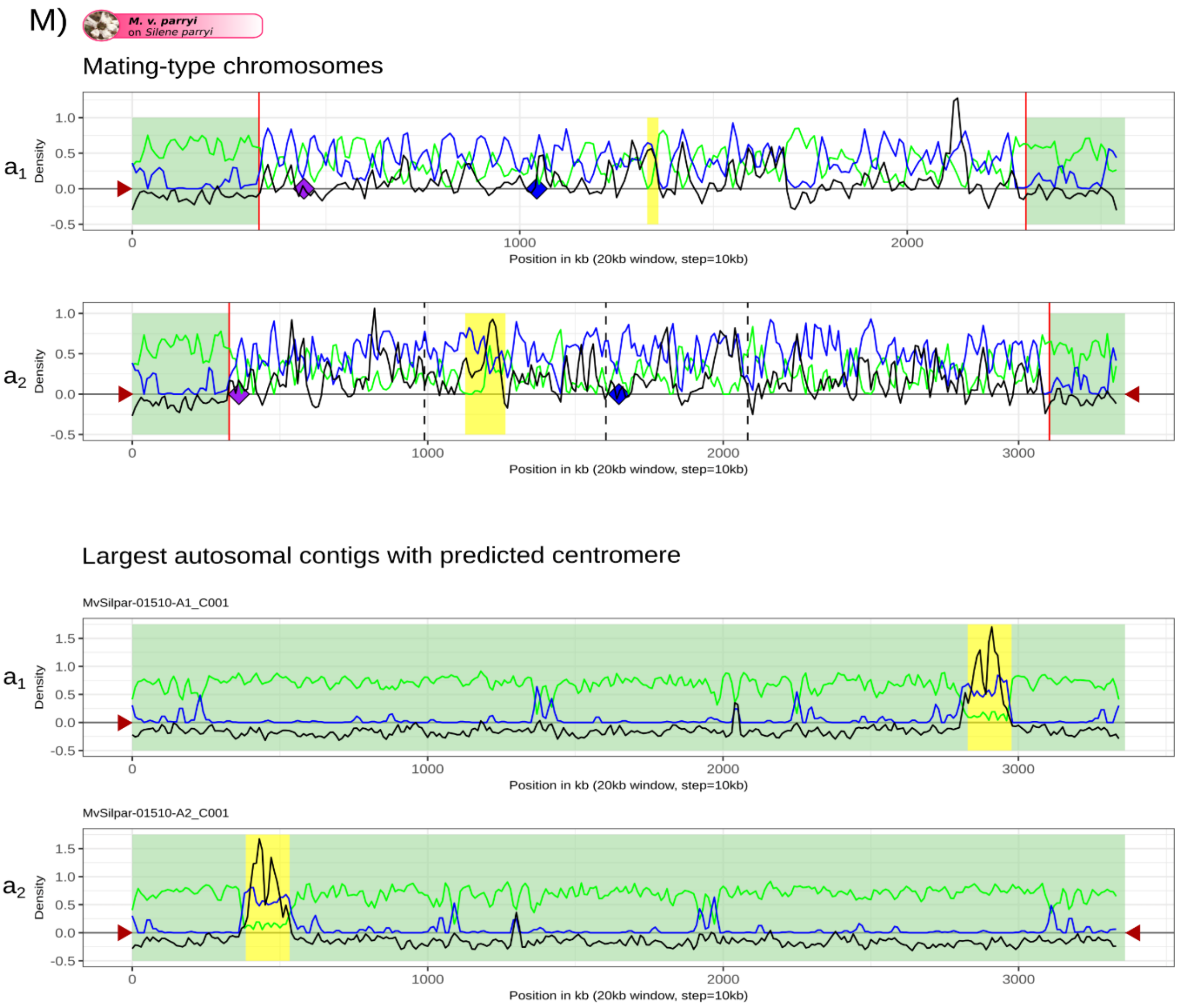

Key to lines
- Transposable elements (TEs)
- Genes
- Repeat-induced point mutation (RIP) index

Key to symbols
- PR locus
- HD locus
- Telomere

Key to boundaries
- Non-recombining region (center) / pseudo-autosomal region (flanking)
- Contig junction (not fully assembled mating-type chromosome)

Key to boxes
- Predicted centromere
- Recombining region

N)

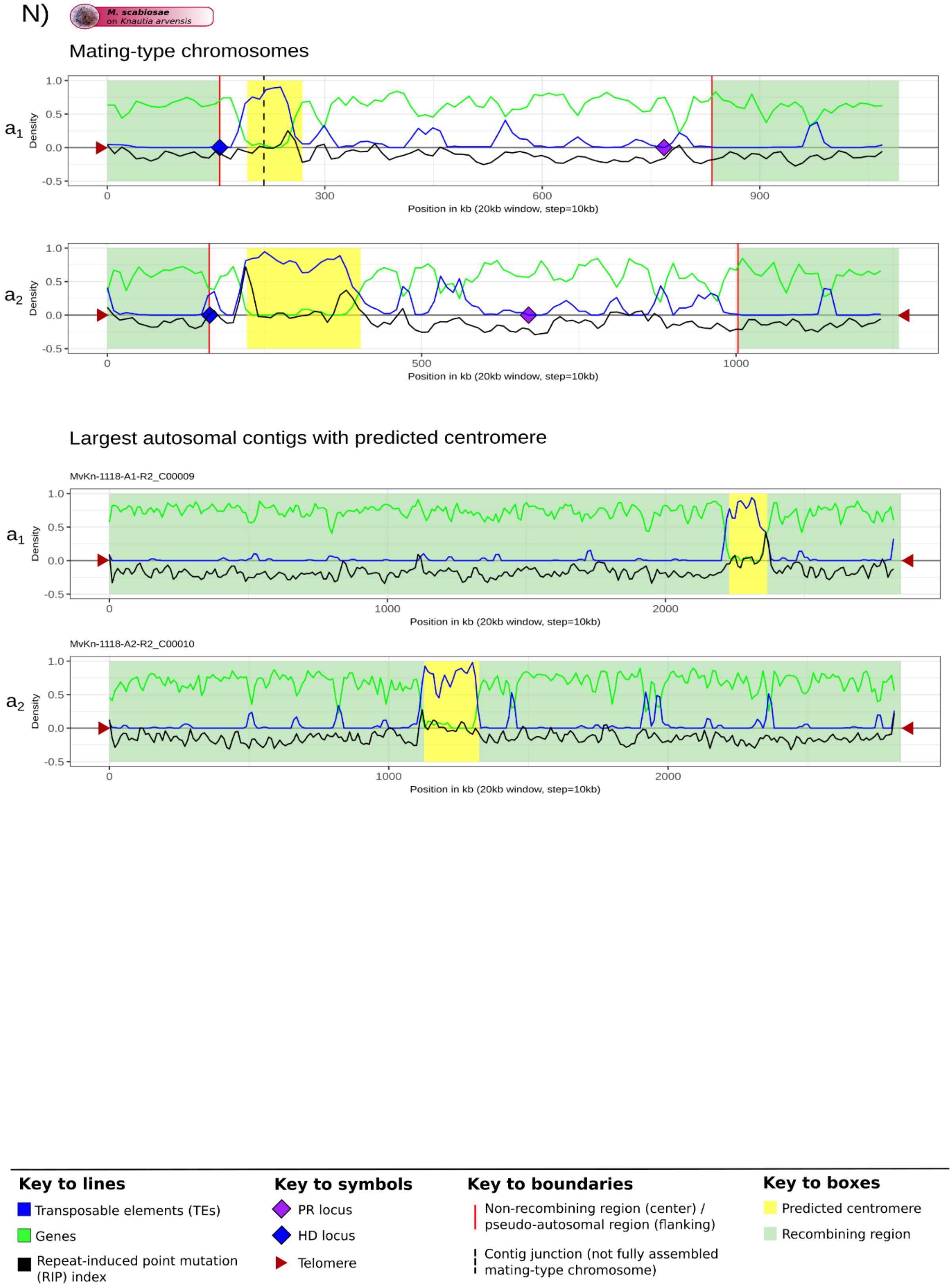
M. scabiosae
on Knautia arvensis
Mating-type chromosomes
a1
a2
Density
Position in kb (20kb window, step=10kb)
Largest autosomal contigs with predicted centromere
MvKn-1118-A1-R2_C00009
MvKn-1118-A2-R2_C00010
Key to lines
Transposable elements (TEs)
Genes
Repeat-induced point mutation (RIP) index
Key to symbols
PR locus
HD locus
Telomere
Key to boundaries
Non-recombining region (center) / pseudo-autosomal region (flanking)
Contig junction (not fully assembled mating-type chromosome)
Key to boxes
Predicted centromere
Recombining region

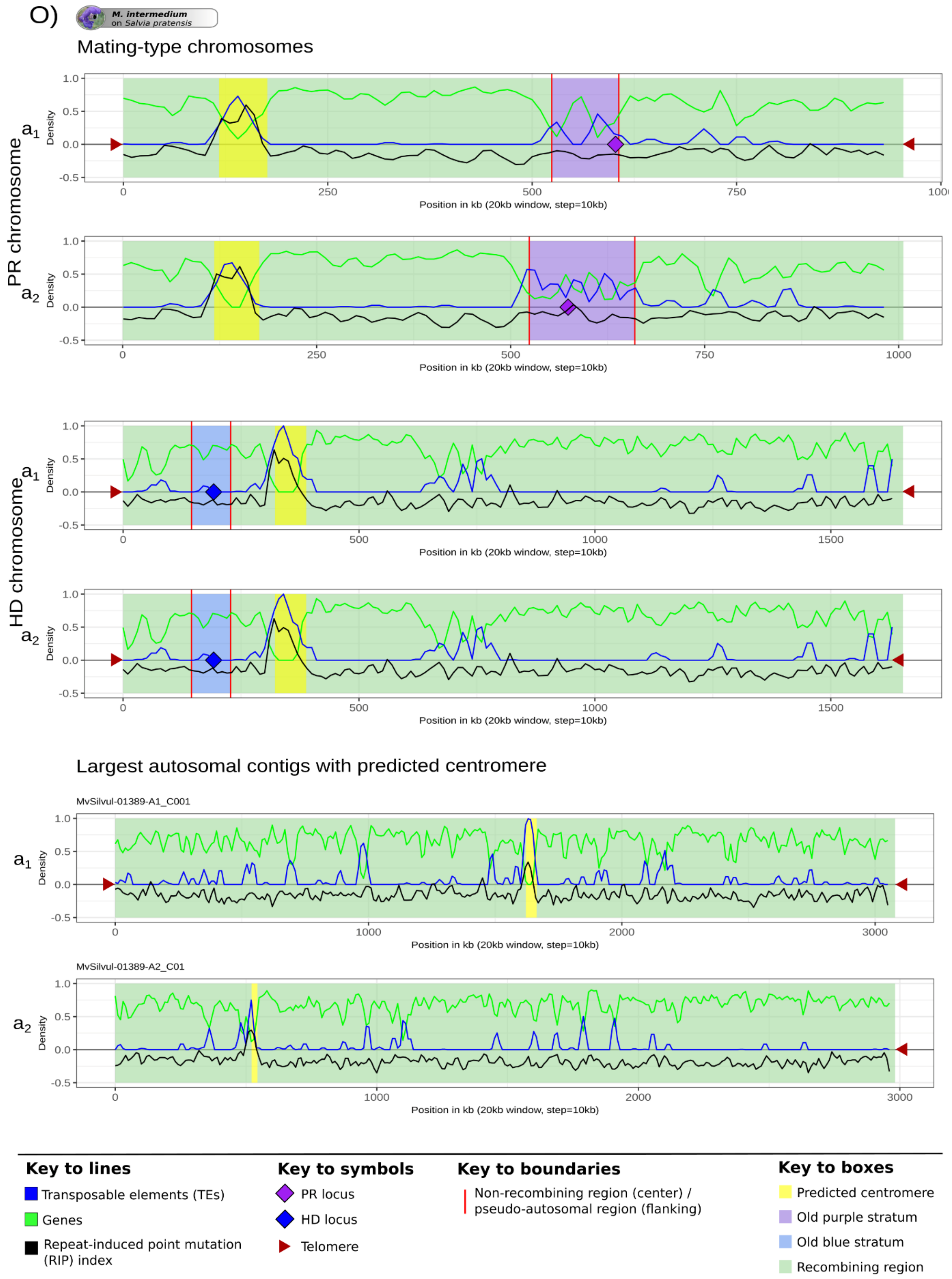

Supplementary Figure 5: Density of transposable elements (TEs), genes and repeat-

induced point mutations (RIP) along the mating-type chromosomes and the autosomes of *Microbotryum* genomes. Density of TEs, genes and RIP index were calculated in 20 kb non-overlapping windows. A RIP index greater than one indicates that the region is RIP-affected. Predicted centromeres are indicated in yellow. Recombining regions of the mating-type chromosomes (pseudo-autosomal regions) and the autosomes (largest autosomal contig harboring a predicted centromere in each genome) are highlighted in pale green. Non-recombining regions are delimited by red lines. Non-rearranged evolutionary strata are displayed in their corresponding color (see legend). Non-fully assembled mating-type chromosomes have been joined and the junctions are indicated by black dashed lines. Pheromone-receptor (*PR*) and homeodomain (*HD*) loci are indicated by purple and blue diamonds, respectively. Identified telomeres are indicated by brown triangles.

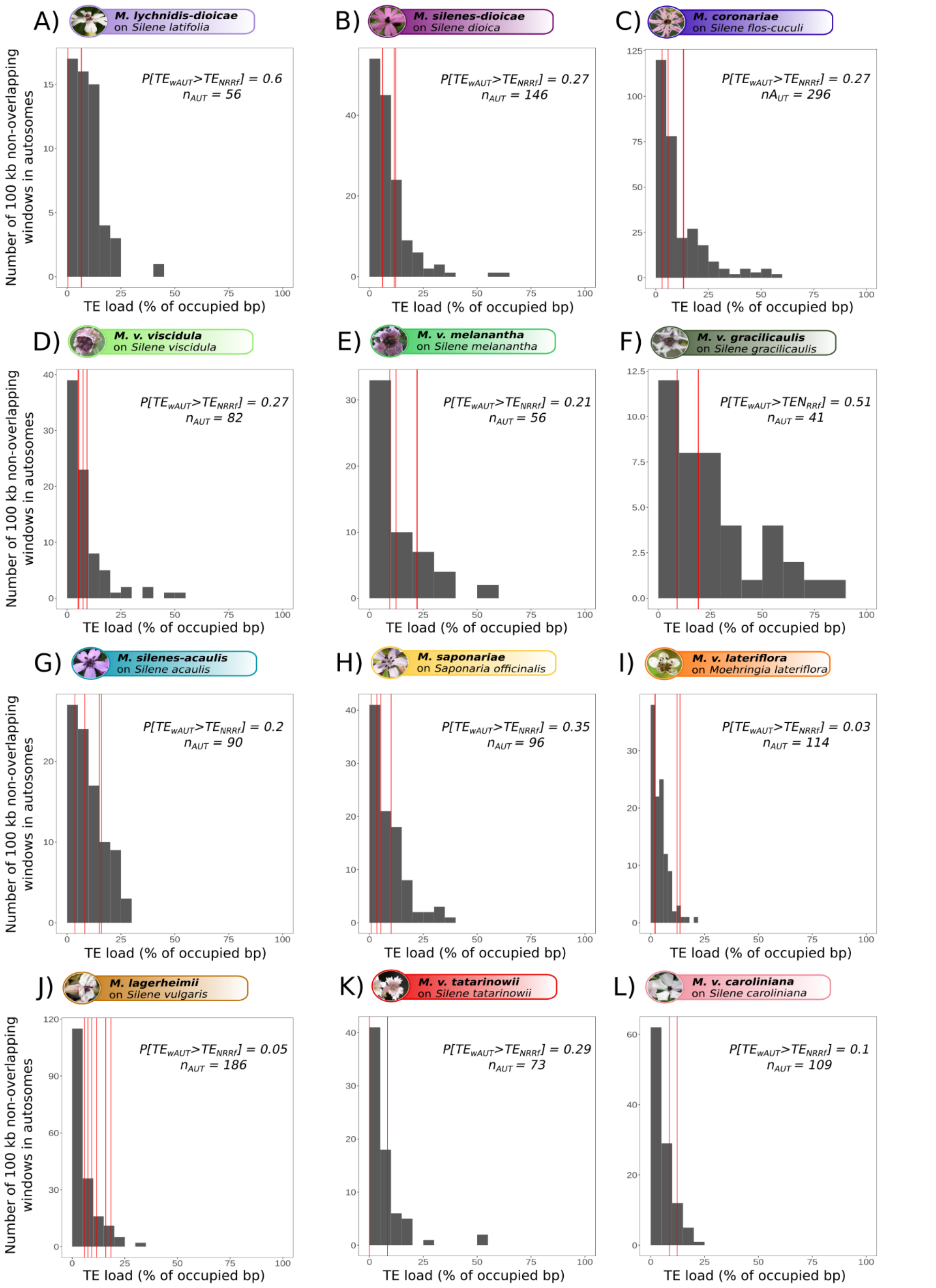

Supplementary Figure 6: Transposable element (TE) load at the margin of the non-

recombining regions compared to the distribution in recombining autosomal regions. The average values of TE content in the regions flanking the non-recombining region are indicated by red lines and the ones in autosomal windows constitute the grey distribution. The probability of finding a higher TE content in a 100 kb window on autosomes than in the 100 kb region directly flanking the non-recombining region ($P[TE_{wAUT}>TE_{NRRf}]$) is reported in the top right corner of each panel. The panels correspond to different species, and there are four or eight red lines (individual flanking regions of non-recombining regions) per species: one a_1 and one a_2 genome per species, and for each haploid genome one or two mating-type chromosomes, so four or eight flanking regions. Three species are not represented because their pseudo-autosomal regions were smaller than 100 kb (*Microbotryum violaceum paradoxa*) or their autosome assemblies were too fragmented (*M. v. parryi* and *M. scabiosae*). The n_{AUT} value corresponds to the number of 100 kb autosomal windows used to calculate the distribution of autosomal TE load.

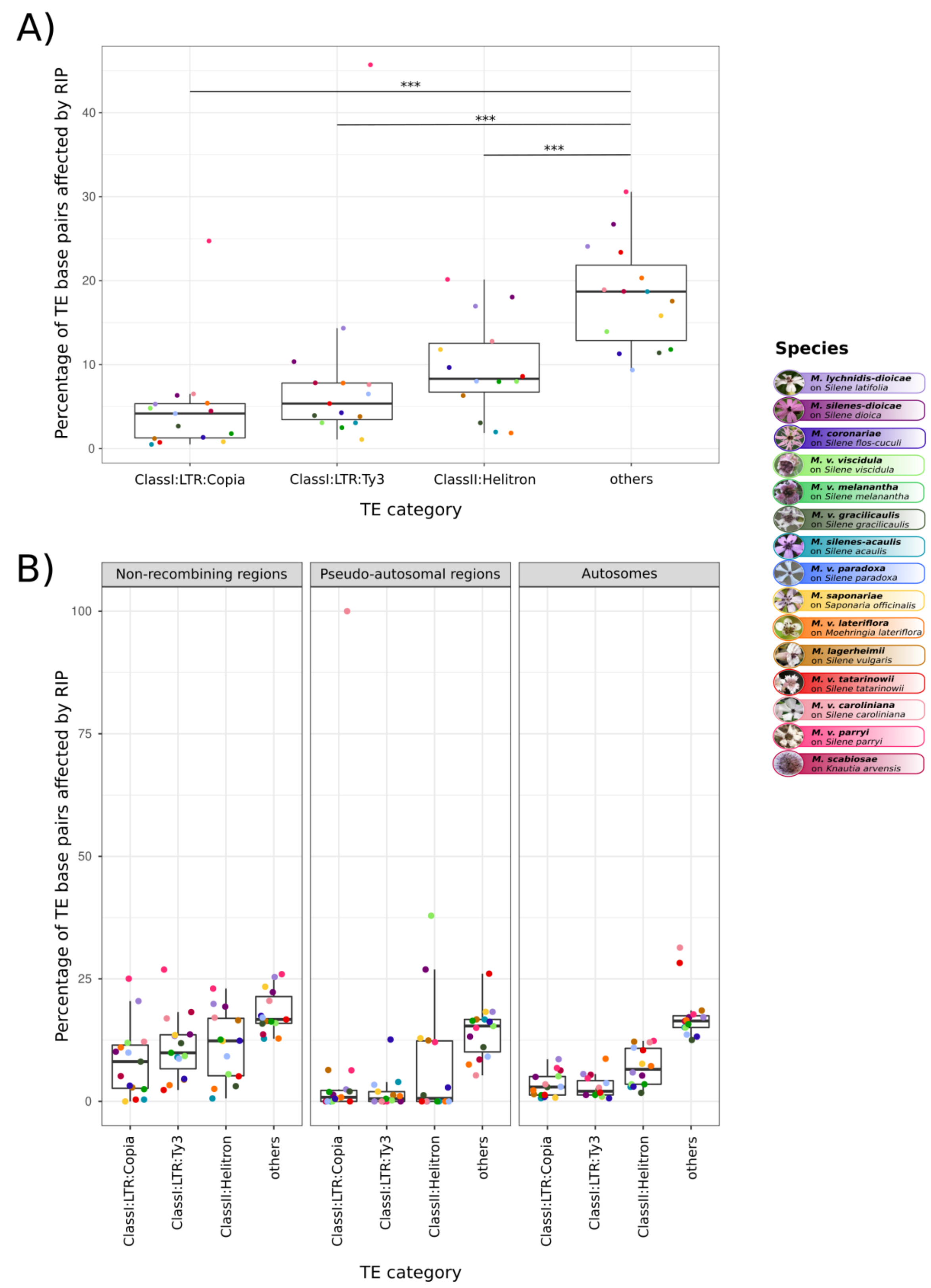

Supplementary Figure 7: Percentage of transposable element (TE) base pairs affected by repeat-induced point mutation (RIP) in *Microbotryum* genomes. Comparison of the proportion of TE base pairs affected by RIP between the TE categories that have expanded in

the non-recombining regions (*Copia*, *Ty3* and *Helitron*) and the other TE categories, genome-wide (A) and across genomic compartments (B). The different colored dots correspond to different *Microbotryum* species. Significant differences between TE categories at the genome scale are indicated by *** (Kolmogorov-Smirnov two-sided test of normality p-value = 0.00001255 and ANOVA post-hoc Tukey test, adj. p-value = 0.0000055, 0.0000006 and 0.0006202 for *Copia Ty3* and *Helitron* vs others, respectively; n = 175 independent percentages of RIP-affected TE base pairs from distinct TE categories and genomic location, see Supplementary Table 2). Boxplots indicate: first quartile Q1 (lower bar of the box), median (line within the box), third quartile Q3 (upper bar of the box), Q1-1.5*IQR (lower whisker) and Q3+1.5*IQR (upper whisker).

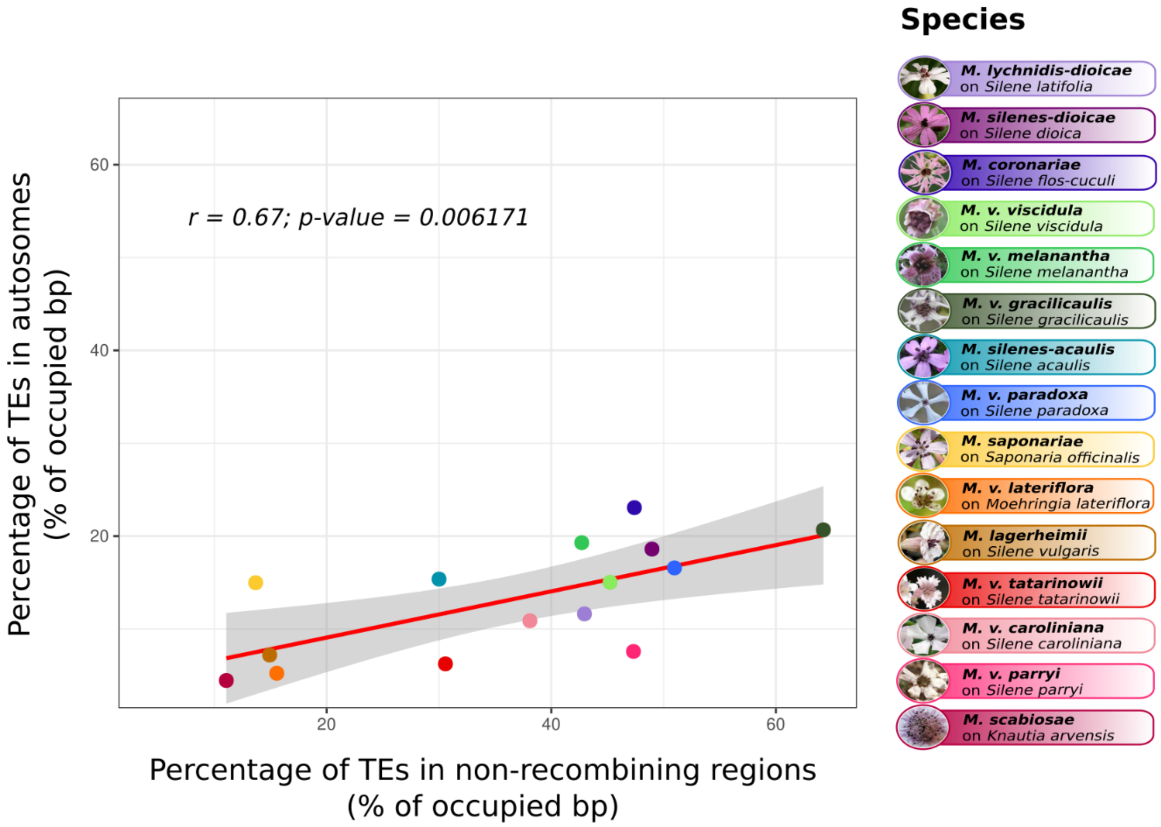

Supplementary Figure 8: Correlation between the proportion of base pairs occupied by transposable elements (TEs) in the non-recombining regions and the autosomes. Significant two-sided Pearson's correlation test, r = 0.67; p-value = 0.006; n = 15. Simple linear

regression is shown as a red line and the 95% confidence intervals of the prediction as grey area.

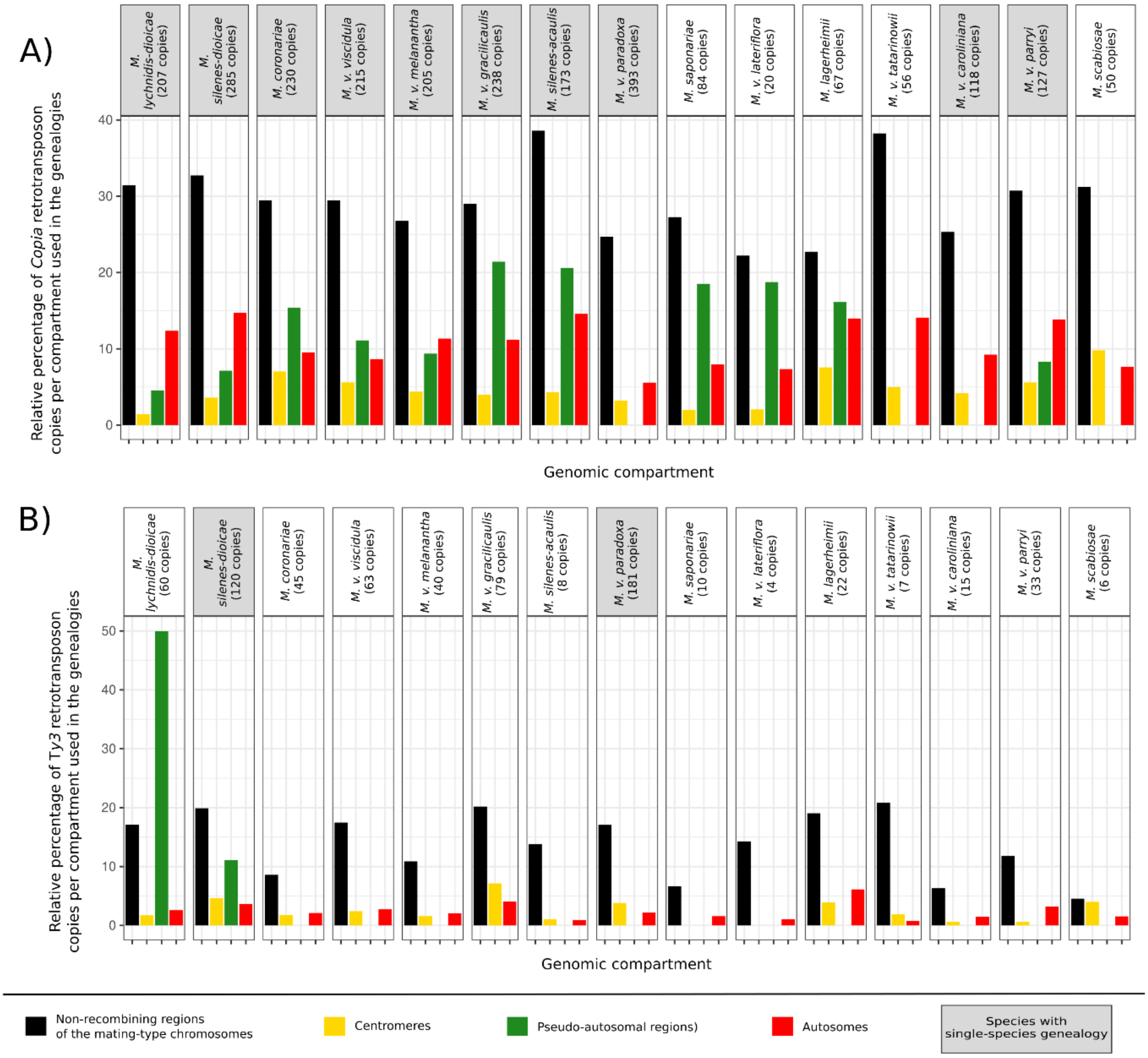

Supplementary Figure 9: Relative percentage and number of transposable element (TE) sequences per genomic compartment used for the genealogies based on their 5'-LTR sequences, for *Copia* (A) and *Ty3* (B) elements. The total number of 5'-LTR sequences available per species is indicated in brackets under the species name. Single-species genealogies were only built for species with grey filled rectangles around their names.

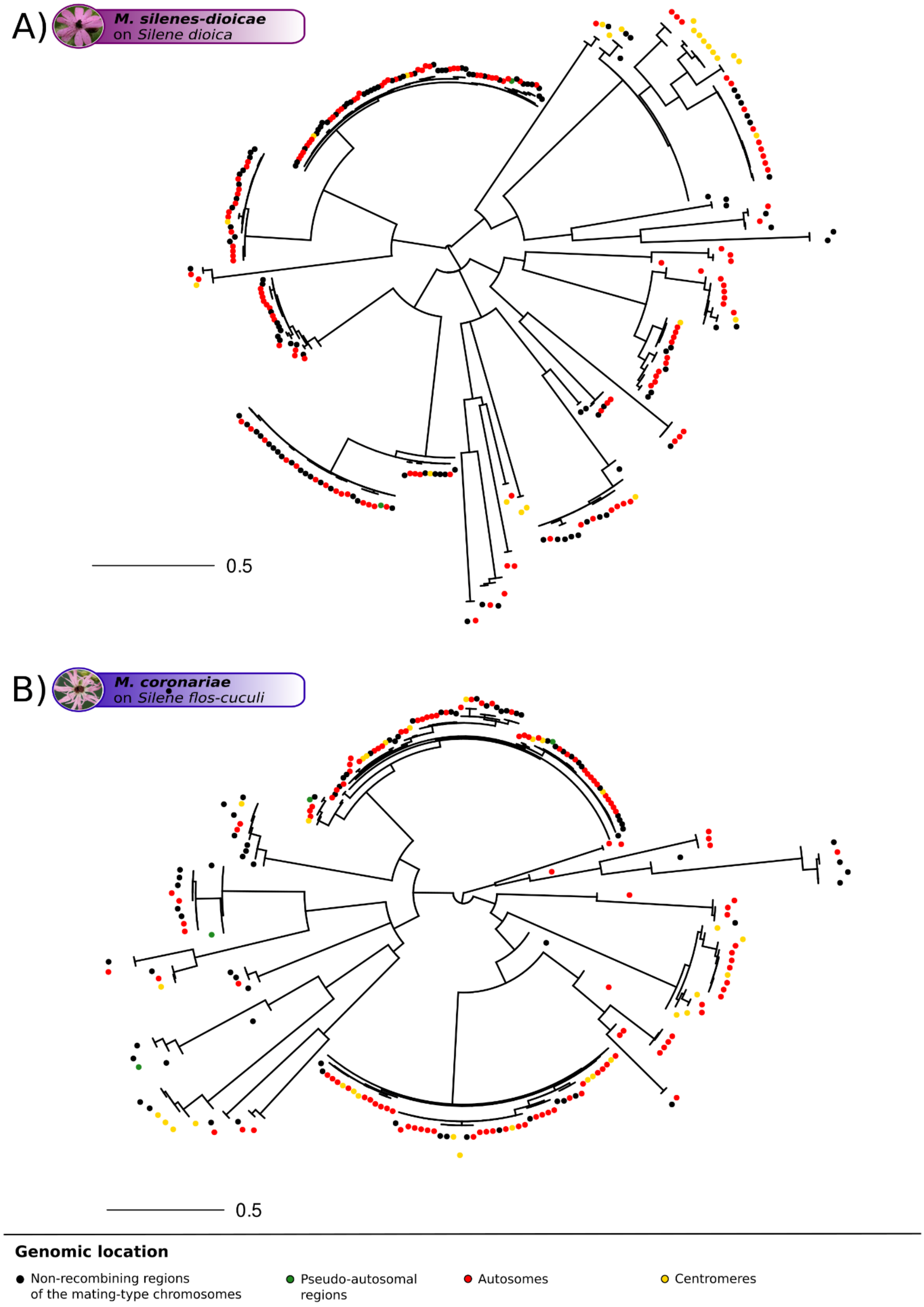
A)
M. silenes-dioicae
on Silene dioica
0.5
B)
M. coronariae
on Silene flos-cuculi
0.5
Genomic location
Non-recombining regions of the mating-type chromosomes
Pseudo-autosomal regions
Autosomes
Centromeres

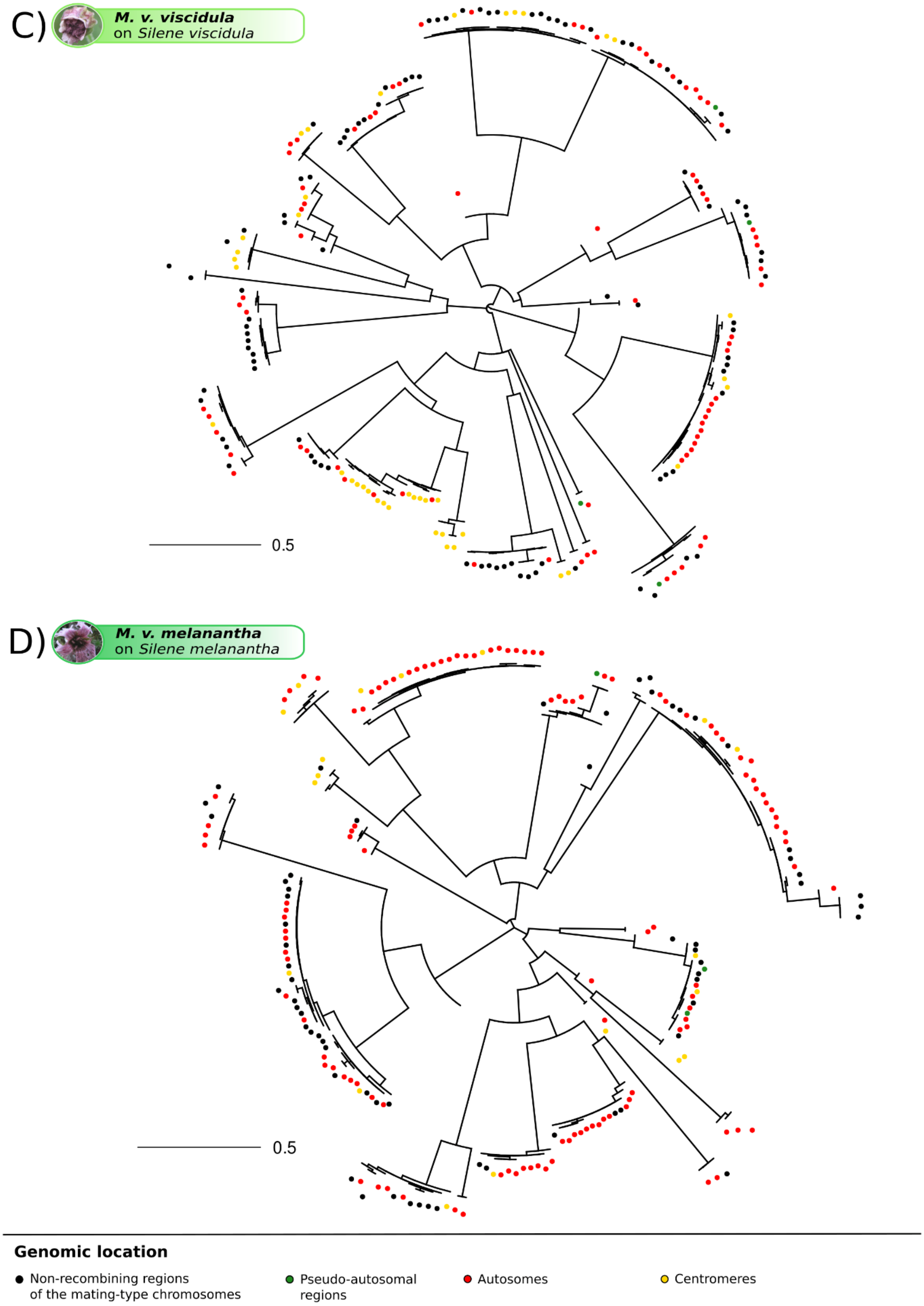
C)
M. v. viscidula
on Silene viscidula
0.5
D)
M. v. melanantha
on Silene melanantha
0.5
Genomic location
Non-recombining regions of the mating-type chromosomes
Pseudo-autosomal regions
Autosomes
Centromeres

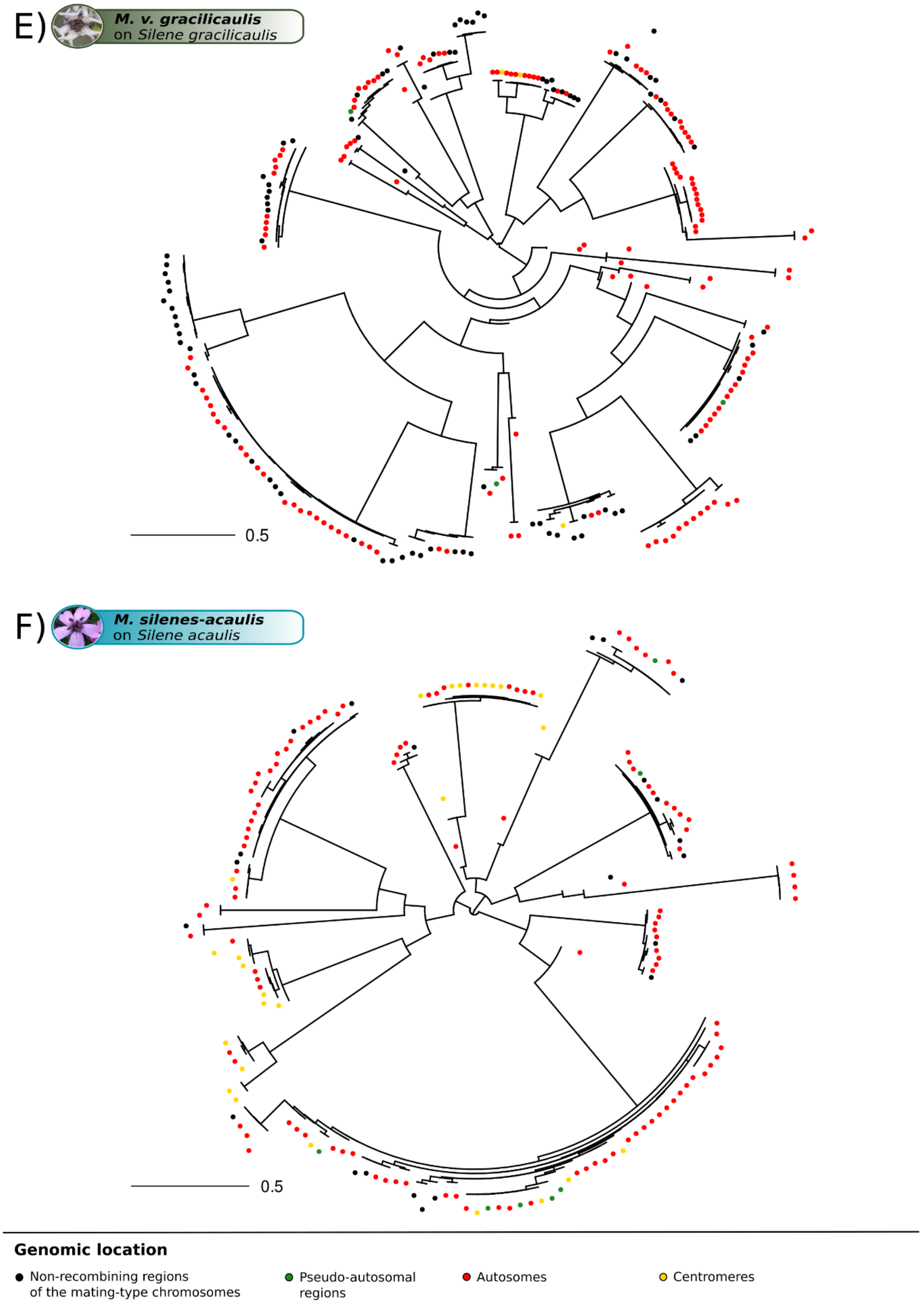
E)
M. v. gracilicaulis
on Silene gracilicaulis
0.5
F)
M. silenes-acaulis
on Silene acaulis
0.5
Genomic location
Non-recombining regions of the mating-type chromosomes
Pseudo-autosomal regions
Autosomes
Centromeres

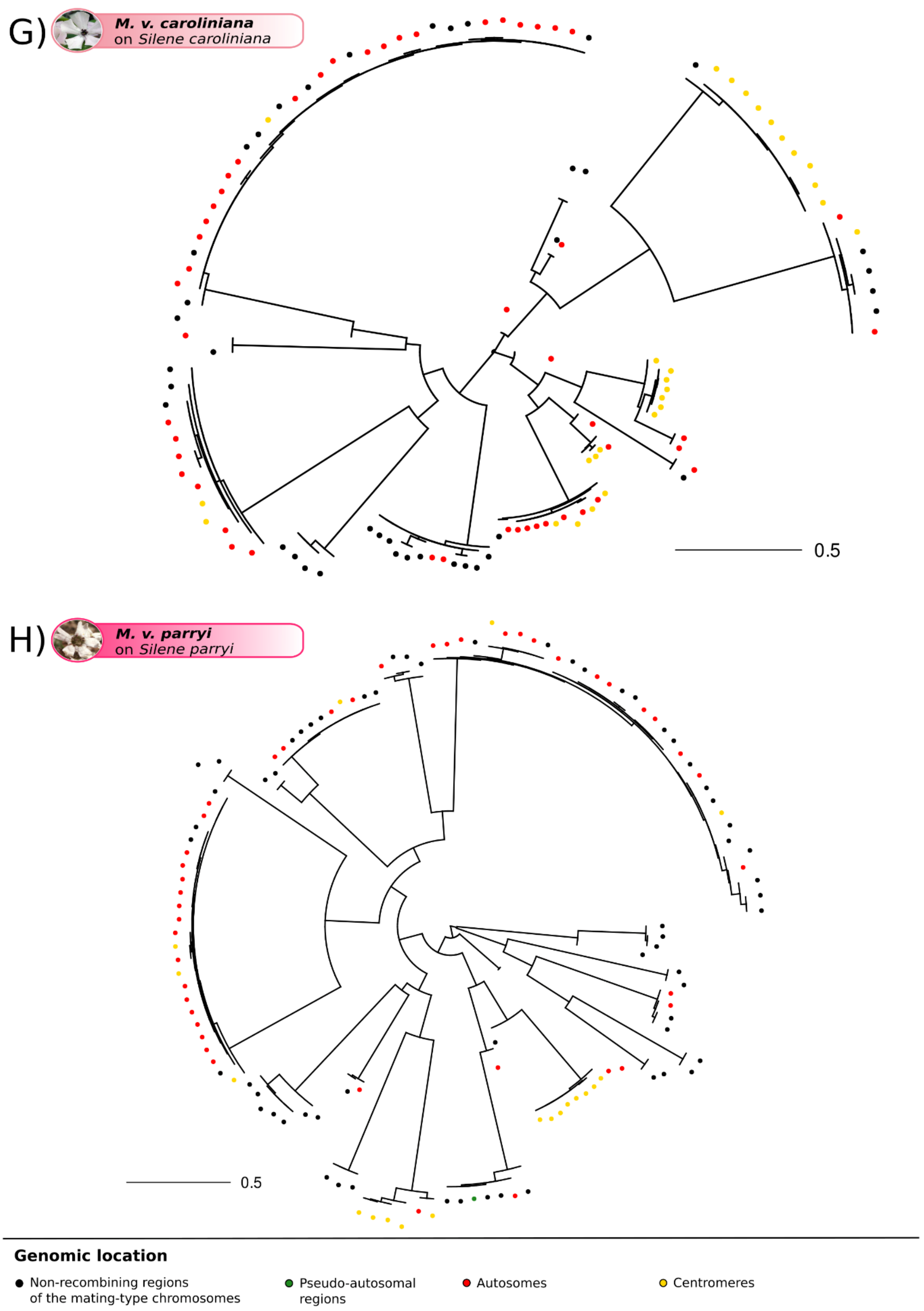

Supplementary Figure 10: Single-species genealogies of *Copia* retroelement copies in

***Microbotryum* genomes based on long-tandem repeat (LTR) sequences.** Single-species genealogies of *Copia* copies in *Microbotryum silenes-dioicae* **(A)**, *M. coronariae* **(B)**, *M. violaceum viscidula* **(C)**, *M. v. melanantha* **(D)**, *M. v. gracilicaulis* **(E)**, *M. silenes-acaulis* **(F)**, *M. v. caroliniana* **(G)** and *M. v. parryi* **(H)**. The color of the dots at the tip of the branches corresponds to the genomic location of the transposable element (TE) copies.

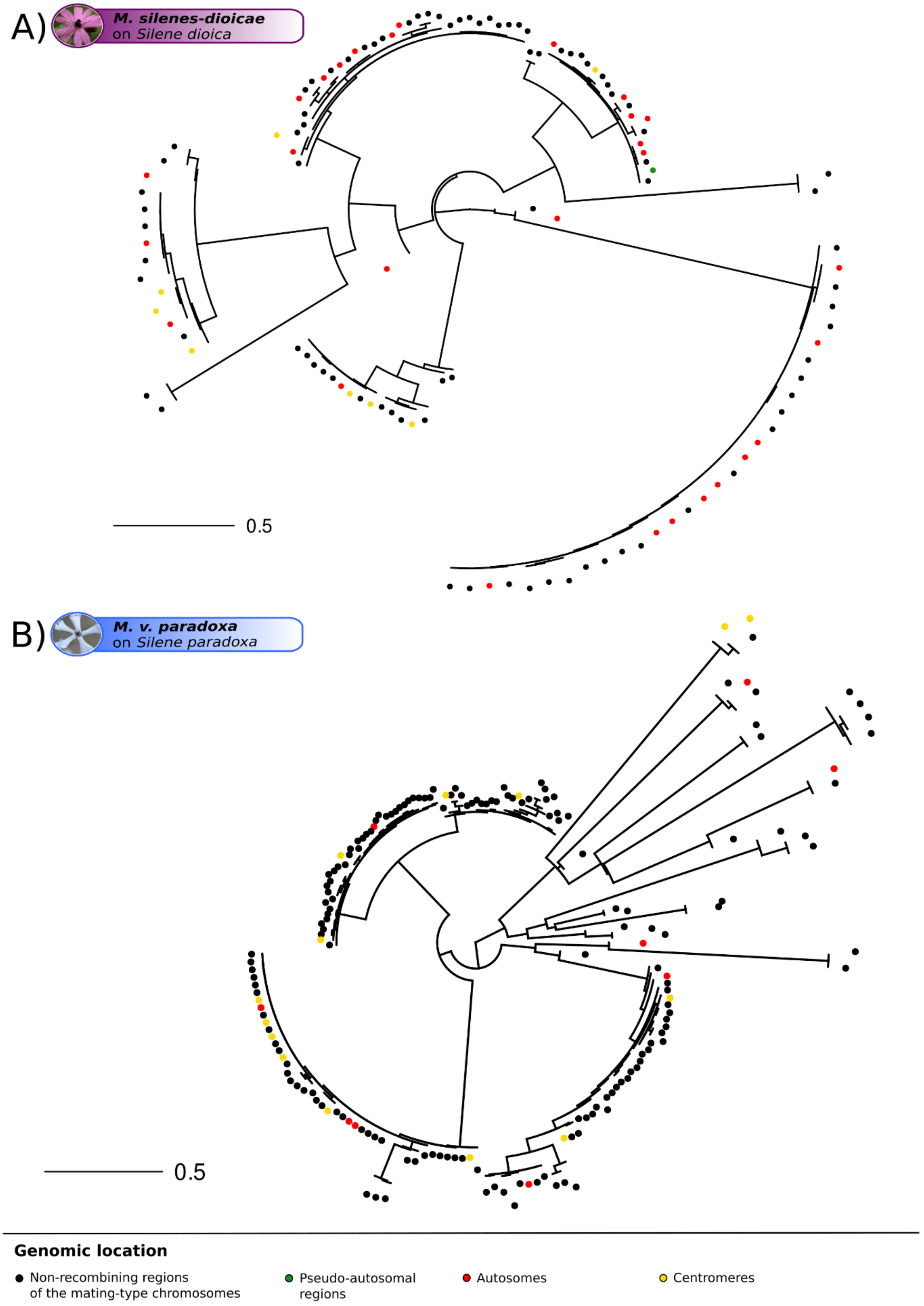
A)
M. silenes-dioicae
on Silene dioica
0.5
B)
M. v. paradoxa
on Silene paradoxa
0.5
Genomic location
Non-recombining regions of the mating-type chromosomes
Pseudo-autosomal regions
Autosomes
Centromeres

Supplementary Figure 11: Single-species genealogies of *Ty3* retroelement copies in *Microbotryum* genomes based on long-tandem repeat (LTR) sequences. Single-species genealogies of *Ty3* copies in *Microbotryum silenes-dioicae* **(A)** and *M. v. paradoxa* **(B)**. The color of the dots at the tip of the branches corresponds to the genomic location of the transposable element (TE) copies.

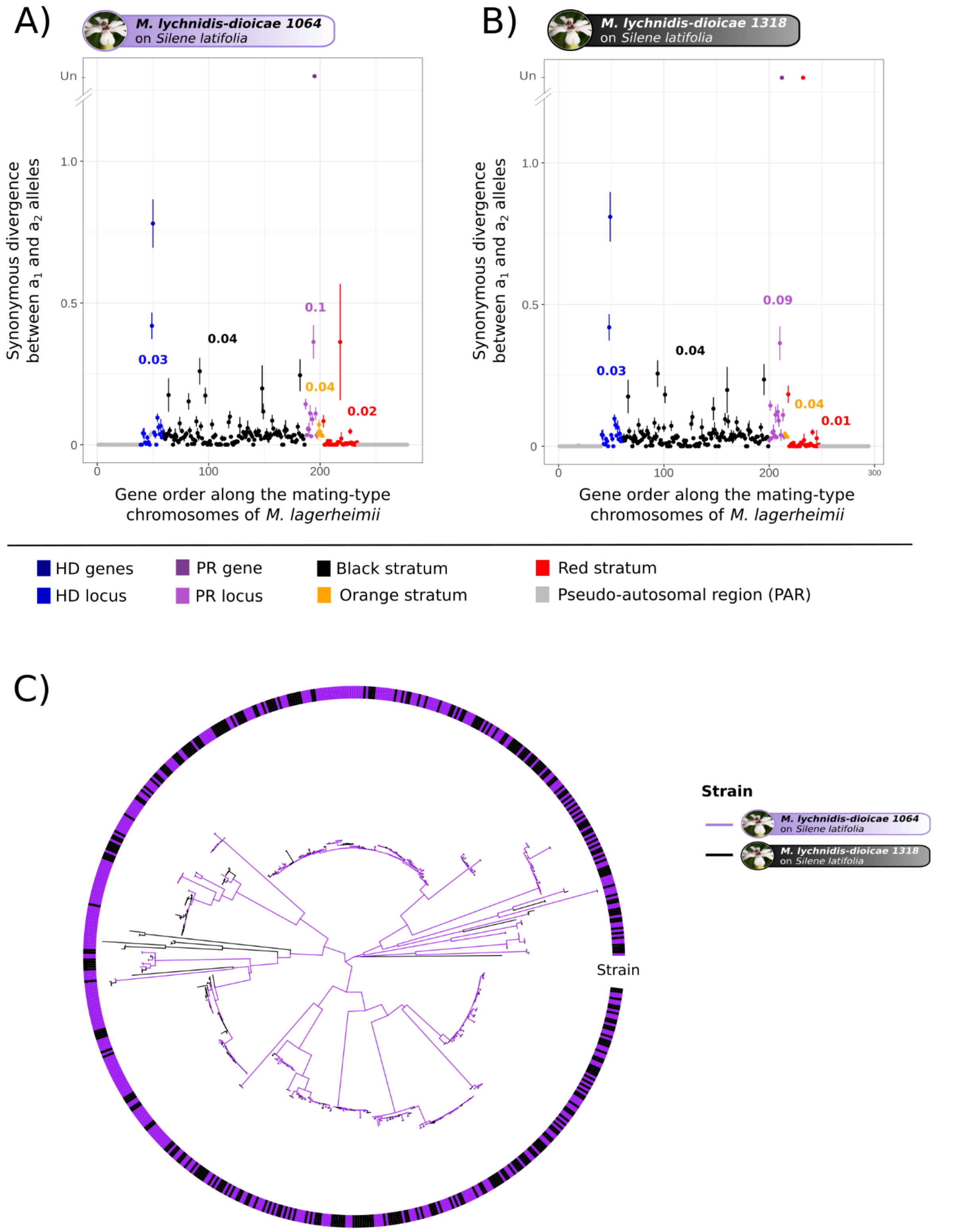

Supplementary Figure 12: Comparison of *Copia* retroelement dynamics in two strains of *Microbotyum lychnidis-dioicae* sharing the same evolutionary strata. Per-gene synonymous

divergence and standard error between a_1 and a_2 alleles on the mating-type chromosomes of the **(A)** 1064 strain and **(B)** 1318 strain of *M. lychnidis-diociae*, plotted along the ancestral gene order inferred from *M. lagerheimii* with unlinked mating-type loci. Synonymous divergence is used as a proxy for time since recombination suppression and the average synonymous divergence value is indicated in the color corresponding to the respective stratum. The purple and blue evolutionary strata were formed around each of the mating-type genes controlling mating compatibility (pheromone receptor, *PR*, in dark purple, and homeodomain, *HD*, in dark blue) and are ancestral in the *Microbotryum* clade. The mating locus linkage event generated the black stratum. Further extension of recombination suppression beyond the mating-type loci generated the younger orange and red evolutionary strata. The recombining regions of the mating-type chromosomes with null synonymous divergence called pseudo-autosomal regions are represented in grey. **(C)** Genealogy of the 1064 and 1318 strains, based on long tandem repeat (LTR) sequences of their *Copia* elements. Terminal branch color and track color corresponds to the *M. lychnidis-diociae* strain. Standard errors were calculated per gene by PAML using the curvature method, *i.e.*, by inverting the matrix of second derivatives of the log-likelihood.

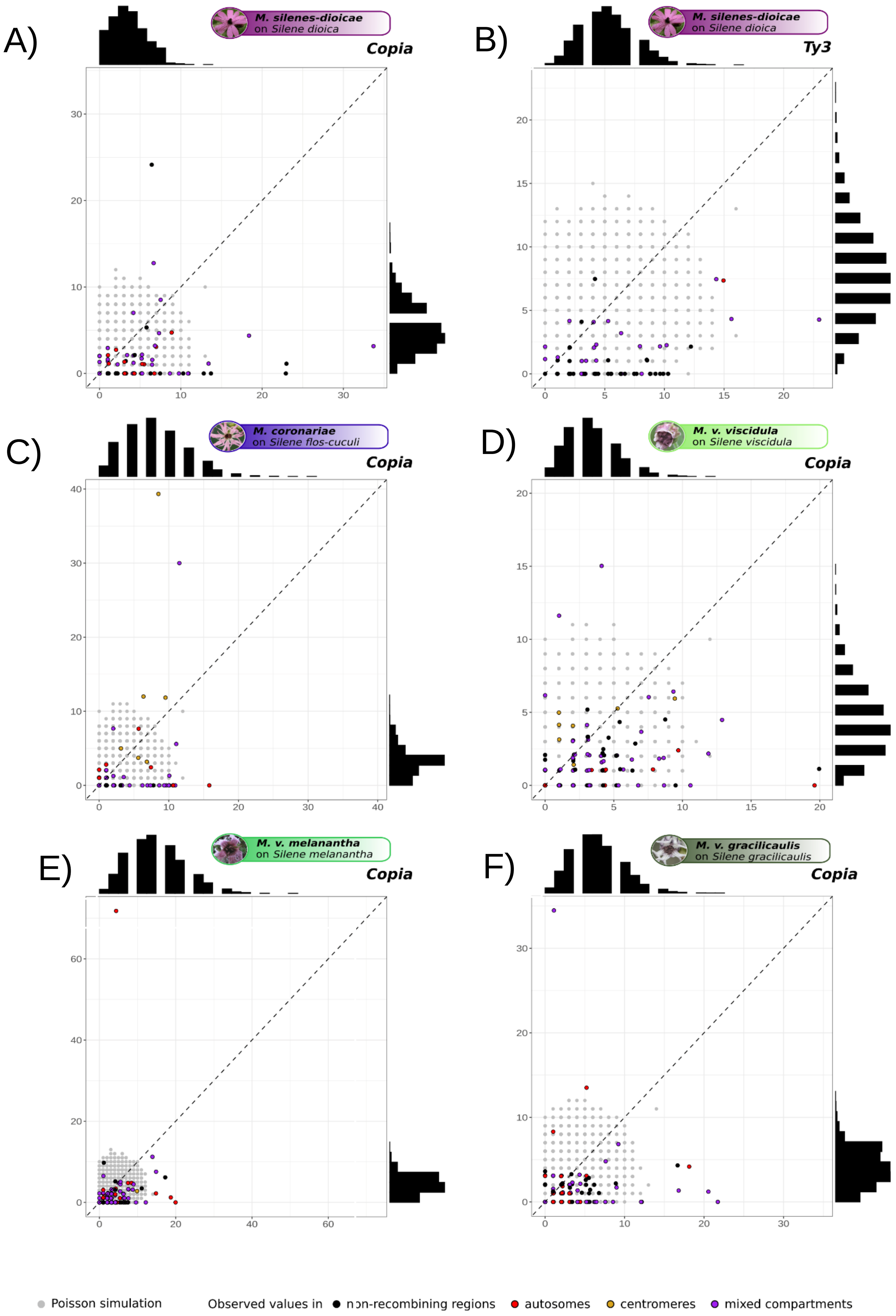
A)
M. silenes-dioicae
on Silene dioica
Copia
B)
M. silenes-dioicae
on Silene dioica
Ty3
C)
M. coronariae
on Silene flos-cuculi
Copia
D)
M. v. viscidula
on Silene viscidula
Copia
E)
M. v. melanantha
on Silene melanantha
Copia
F)
M. v. gracilicaulis
on Silene gracilicaulis
Copia
Poisson simulation
Observed values in
non-recombining regions
autosomes
centromeres
mixed compartments

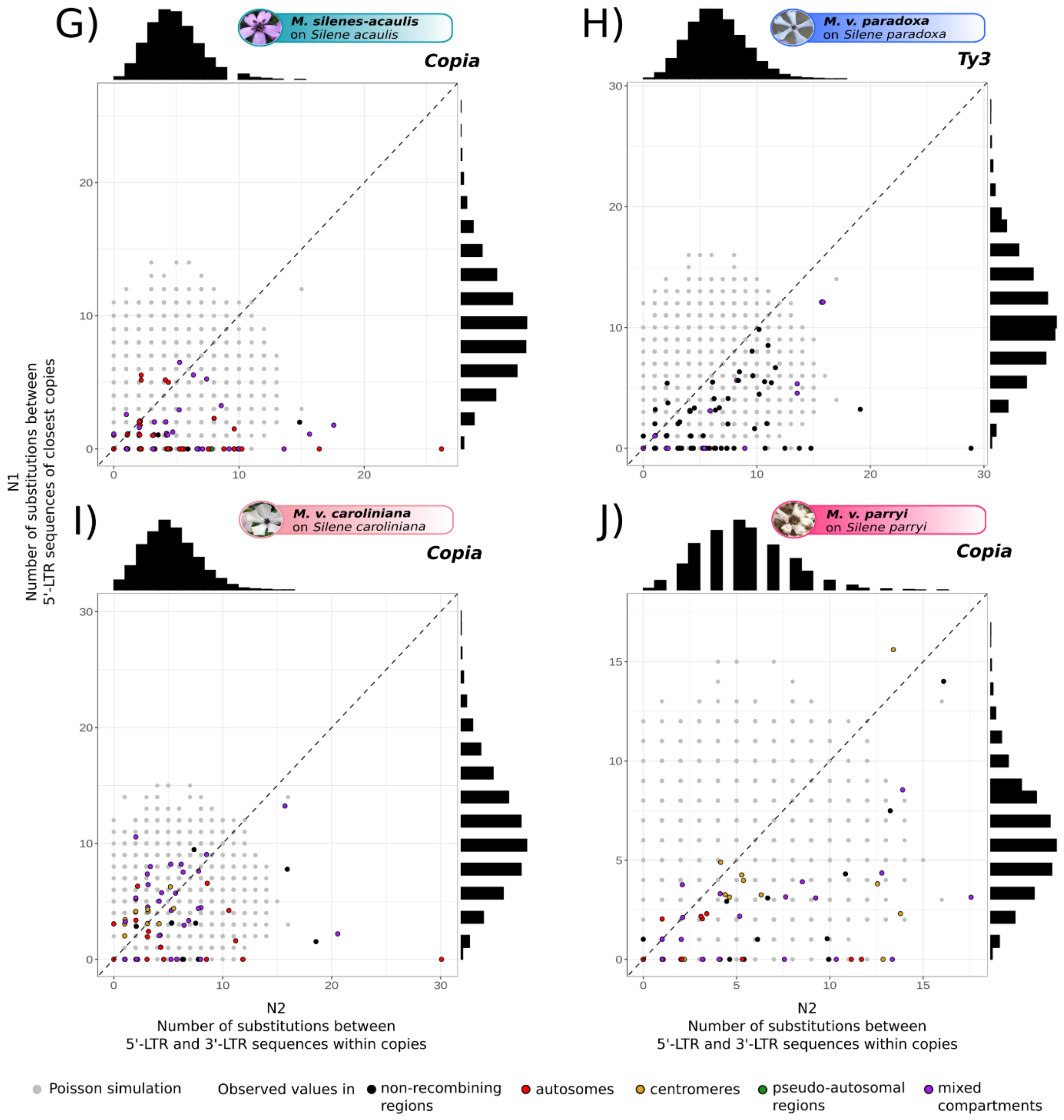

Supplementary Figure 13: Investigation of the possible occurrence of conversion events within clusters of highly similar LTR-retrotransposon copies in *Microbotryum* fungi. We looked for footprints of conversion events as explained on Fig. 5 and in the method section, for the *Copia* **(A)** and *Ty3* **(B)** retrotransposon copies of *Microbotryum silenes-dioicae*, for the *Copia* retrotransposons of *M. coronariae* **(C)**, *M. violaceum viscidula* **(D)**, *M. v. melanantha* **(E)**, *M. v. gracilicaulis* **(F)**, *M. silenes-acaulis* **(G)**, for the *Ty3* retrotransposon copies of *M. v. paradoxa* **(H)**, and for the *Copia* retrotransposon copies of *M. v. caroliniana* **(I)** and *M. v. parryi*

(J). In colors are shown the number of substitutions between the 5'-LTR sequences of pairs of most similar copies (N2) within clusters of highly similar retroelement copies as a function of the number of substitutions between the 5'- and 3'-LTR sequences within copies (N1). The Poisson simulated numbers of substitutions (with lambda equal to the average number of substitutions between the 5'- and 3'-LTR sequences within copies) are plotted in gray. The colors of the points indicate the genomic location of the copy pairs. The observed distributions of the number of substitutions between 5'-LTR sequences and between 5' - 3' LTR sequences are shown at the top and at the right of each scatterplot.

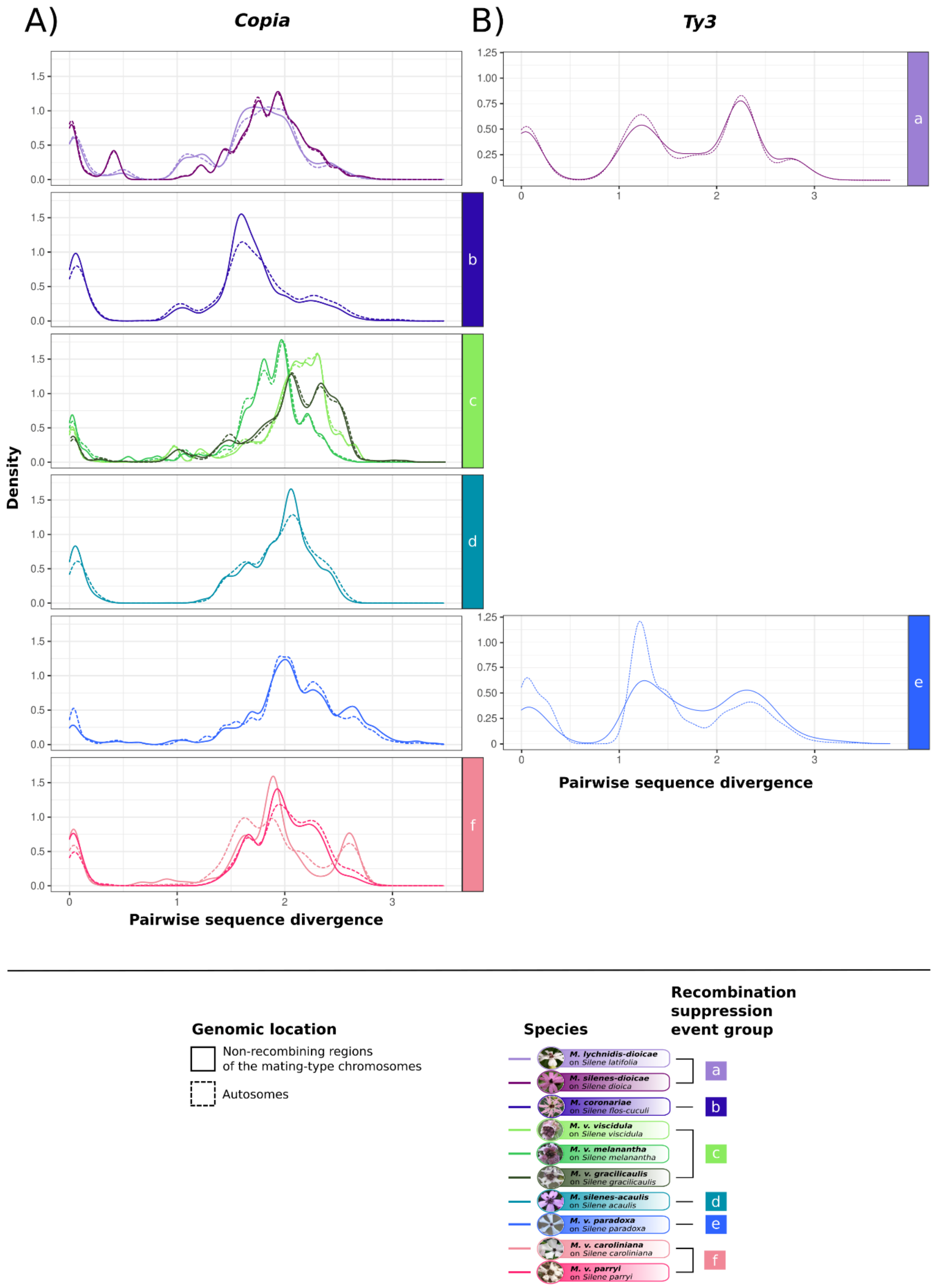

Supplementary Figure 14: Distribution of pairwise divergence for retroelements in the

non-recombining regions of the mating-type chromosomes and the autosomes in each species, plotted separately for the different independent events of recombination suppression. **(A)** *Copia* retrotransposons, **(B)** *Ty3* retrotransposons.

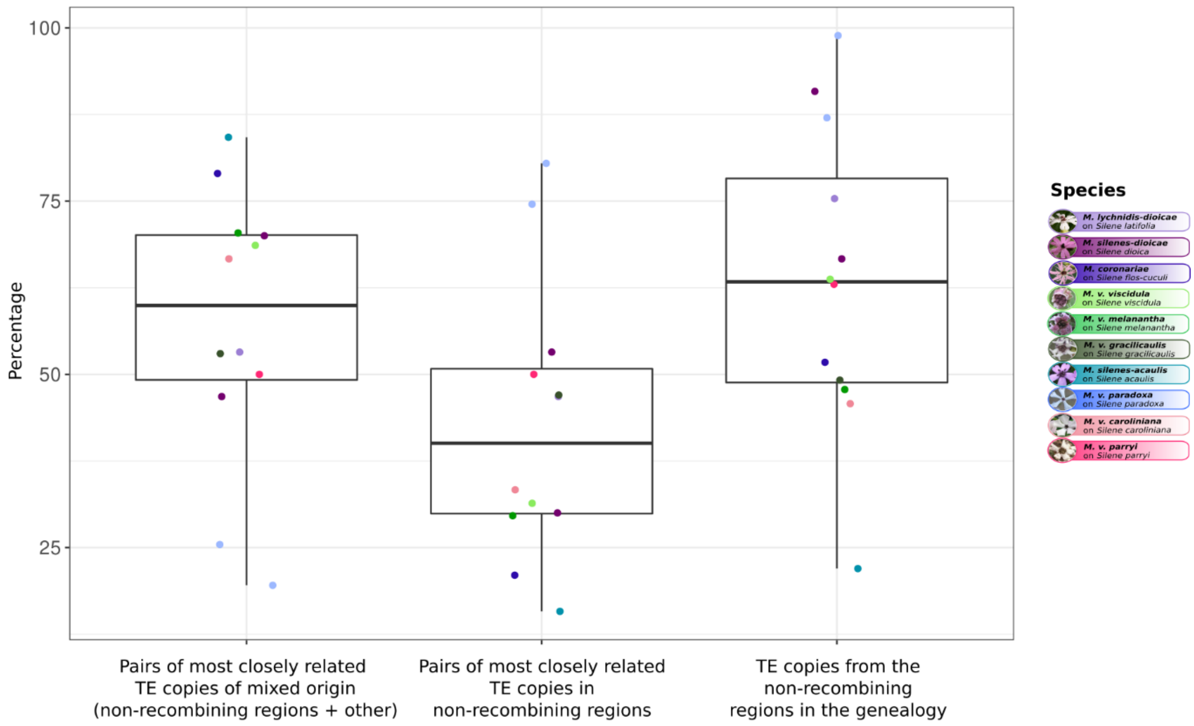

Supplementary Figure 15: Percentage of pairs of most closely related long-terminal repeat (LTR) sequences with at least one copy in the non-recombining regions compared to their representation in their transposable element (TE) genealogy. Each colored point corresponds to a TE genealogy (*Copia* or *Ty3*) of a *Microbotryum* species (Fig. 4 and Supplementary Fig. 10 and 11), according to the species color code from Fig. 1. From left to right are represented the percentages of pairs of most closely related copies i) involving one copy from the non-recombining regions and from another location (*e.g.*, the autosomes), ii) with both copies in the non-recombining regions, and the percentage of copies located in the non-recombining regions, in their TE genealogy.

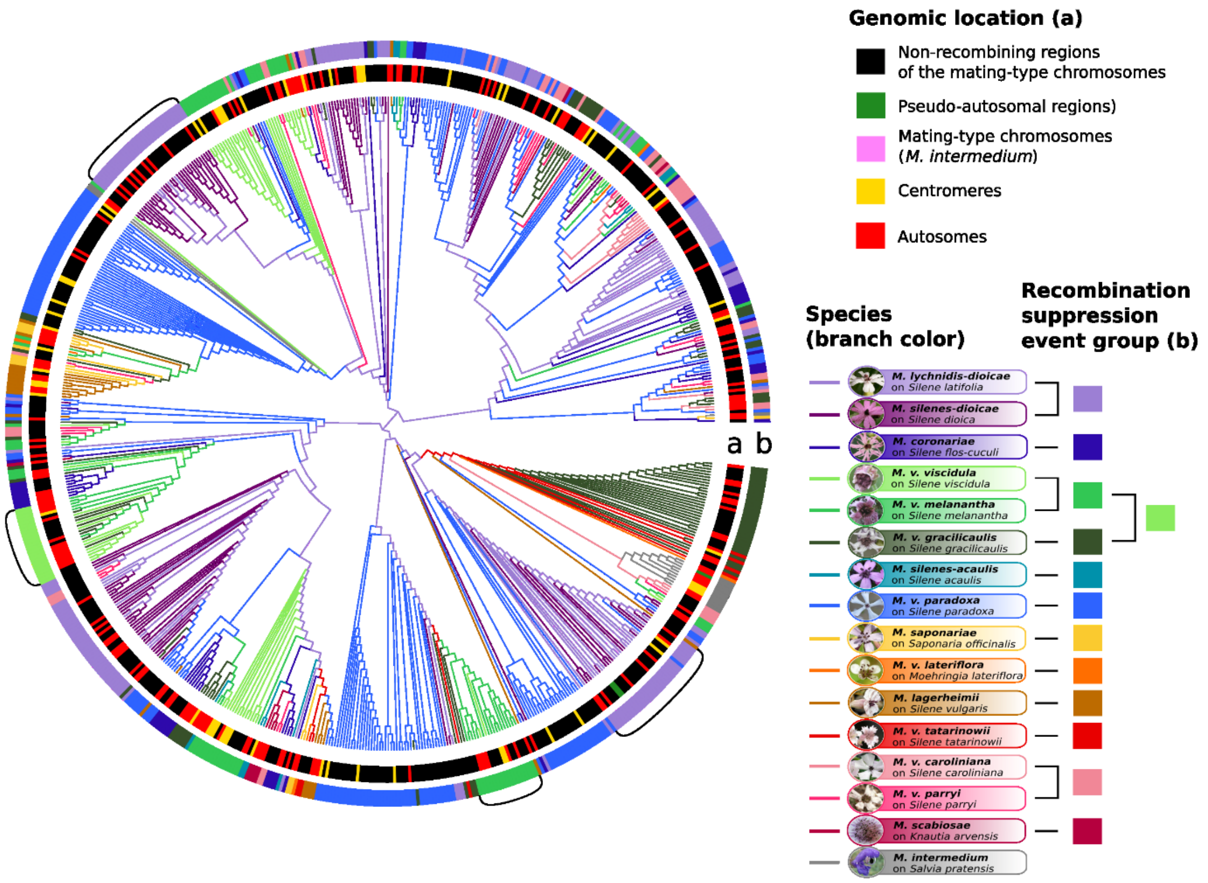

Supplementary Figure 16: Multi-species genealogies of *Ty3* retroelement copies in *Microbotryum* genomes. Multi-species *Ty3* trees of all *Microbotryum* species analysed in this study. The branch color corresponds to the species, the first inner track (a) corresponds to the genomic location of the transposable element (TE) copies, the second outer track (b) corresponds to the linkage event group of the species carrying the TE copy. Brackets highlight bursts of TEs.

A)

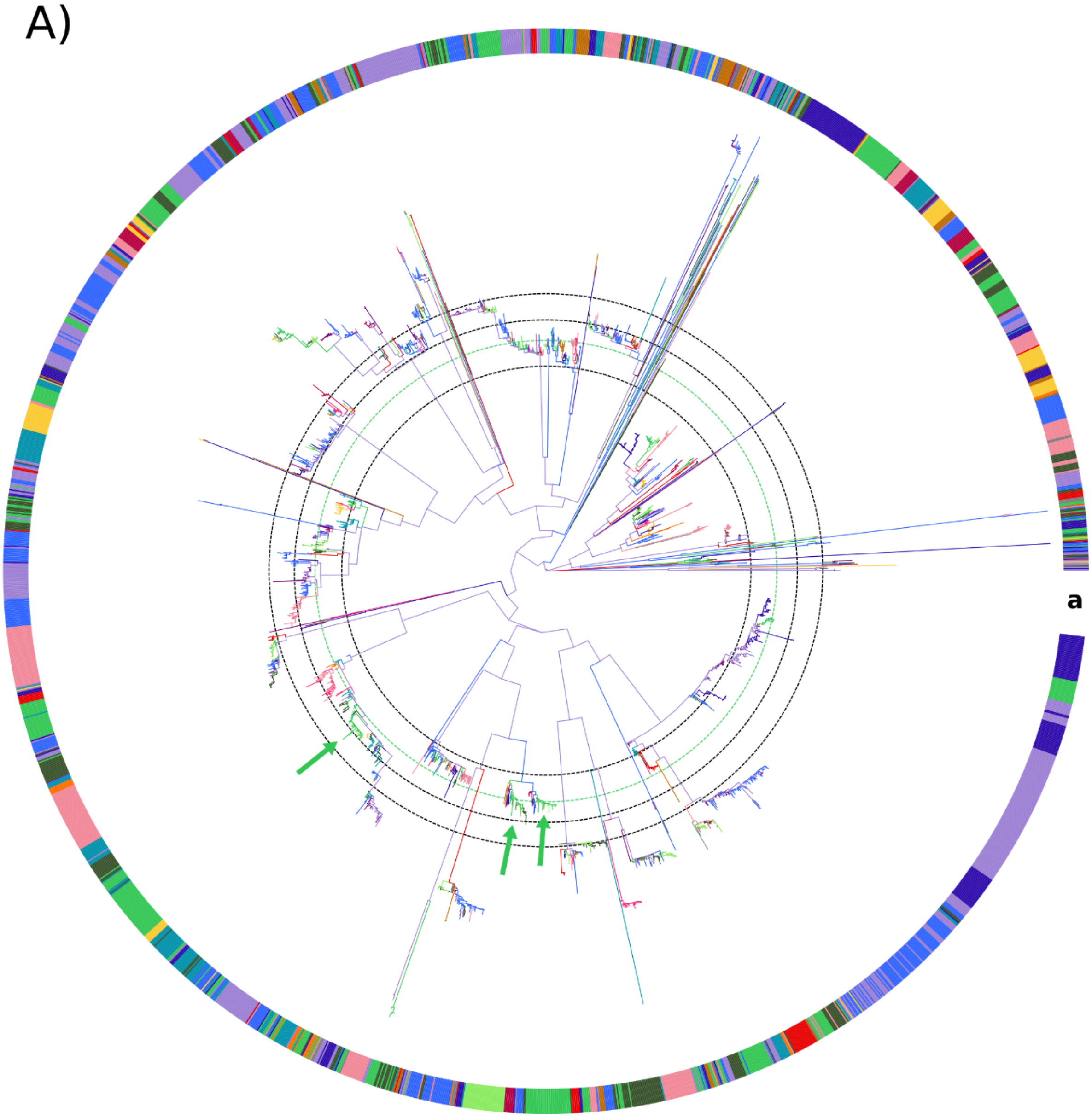

B)

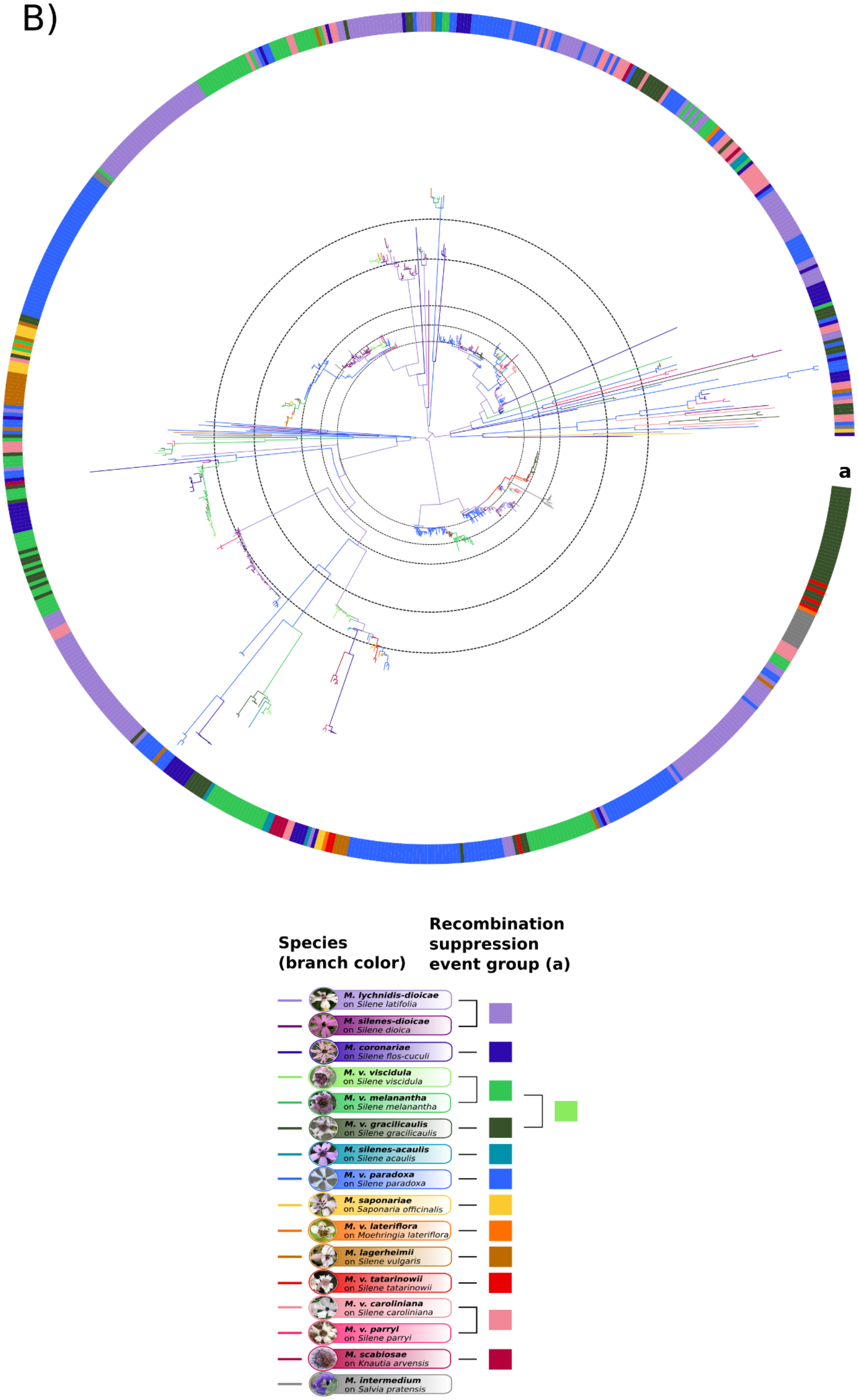
a
Species
(branch color)
Recombination
suppression
event group (a)
M. lychnidis-dioicae
on Silene latifolia
M. silenes-dioicae
on Silene dioica
M. coronariae
on Silene flos-cuculi
M. v. viscidula
on Silene viscidula
M. v. melanantha
on Silene melanantha
M. v. gracilicaulis
on Silene gracilicaulis
M. silenes-acaulis
on Silene acaulis
M. v. paradoxa
on Silene paradoxa
M. saponariae
on Saponaria officinalis
M. v. lateriflora
on Moehringia lateriflora
M. lagerheimii
on Silene vulgaris
M. v. tatarinowii
on Silene tatarinowii
M. v. caroliniana
on Silene caroliniana
M. v. parryi
on Silene parryi
M. scabiosae
on Knautia arvensis
M. intermedium
on Salvia pratensis

Supplementary Figure 17: Multi-species genealogies of retroelement copies in *Microbotryum* genomes based on long-tandem repeat (LTR) sequences. The phylogenies of *Copia* **(A)** and *Ty3* **(B)** are the same as those displayed on Fig. 5 and Supplementary Fig. 16, respectively, but here plotted with branch lengths. The branch color corresponds to the species, the track (a) corresponds to the linkage event group of the species carrying the TE copy. The dashed circles are a help to visualize the timing of the bursts. The green dashed circle highlights the *Copia* bursts which are almost synchronized in the clade of *M. v. gracilicaulis*, pointed by green arrows.

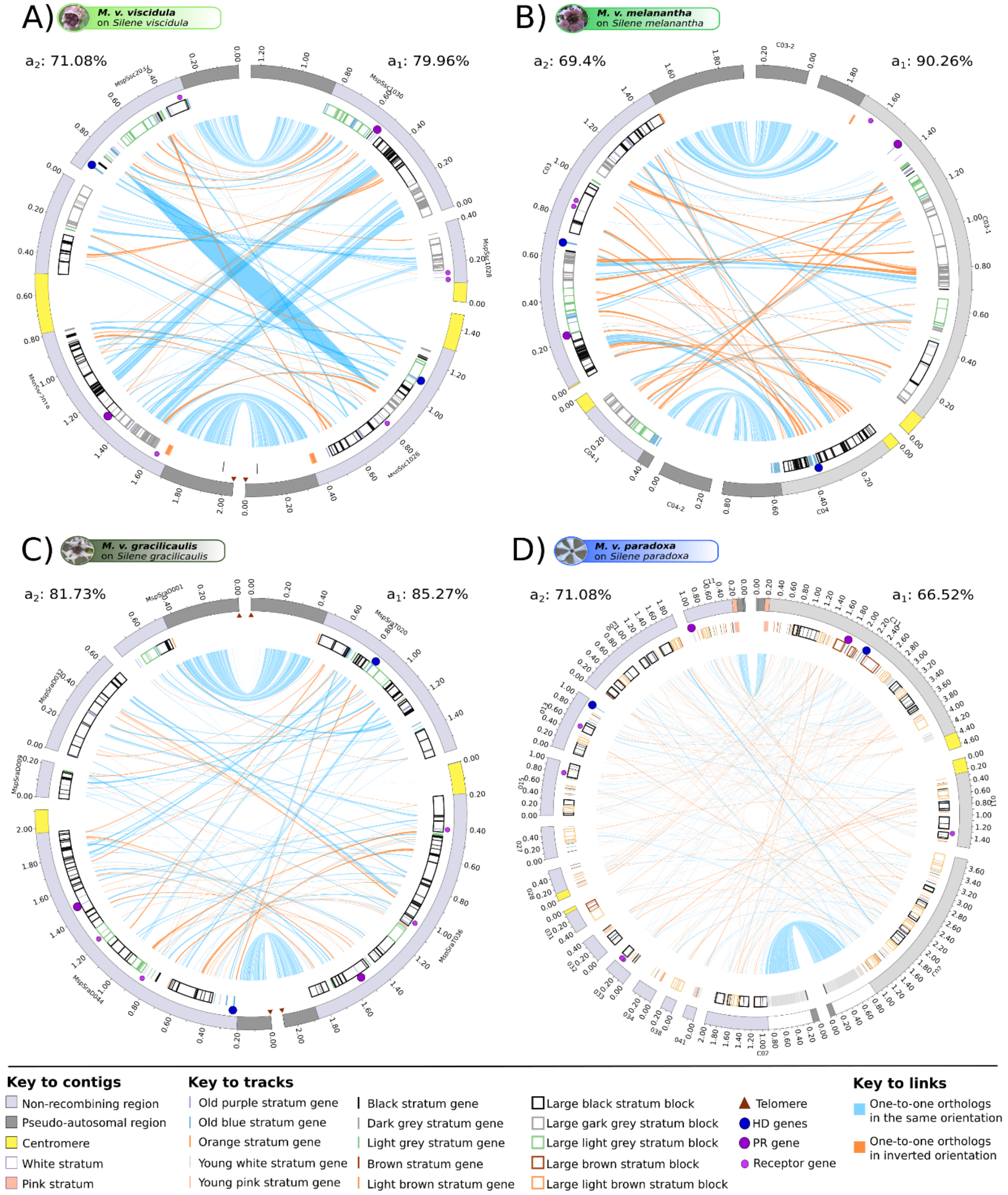

Supplementary Figure 18: Delimitation of blocks for the rearranged evolutionary strata in mating-type chromosomes. The *PR*, *HD* and pheromone genes are represented by purple, blue and small light purple circles, respectively. The outer track represents contigs, with length ticks every 200 kb and centromeres indicated in yellow. Non-recombining regions are displayed

in dark grey and pseudo-autosomal regions in light gray. The young, non-rearranged white and pink strata of *M. v. paradoxa* are highlighted on the outer track. Genes from the different strata are displayed by bars on the inner track (see Fig. 1 for the stratum formation history). Large blocks (greater than 80 kb) of genes from a single stratum are indicated on the inner track by rectangles. The light grey stratum is displayed in green to facilitate visualization. The percentage of non-recombining regions retained in the blocks of strata are indicated next to the mating type, on each side of the circos plots. Blue and orange lines link alleles, the latter corresponding to inversions. The link width is proportional to the corresponding gene length.

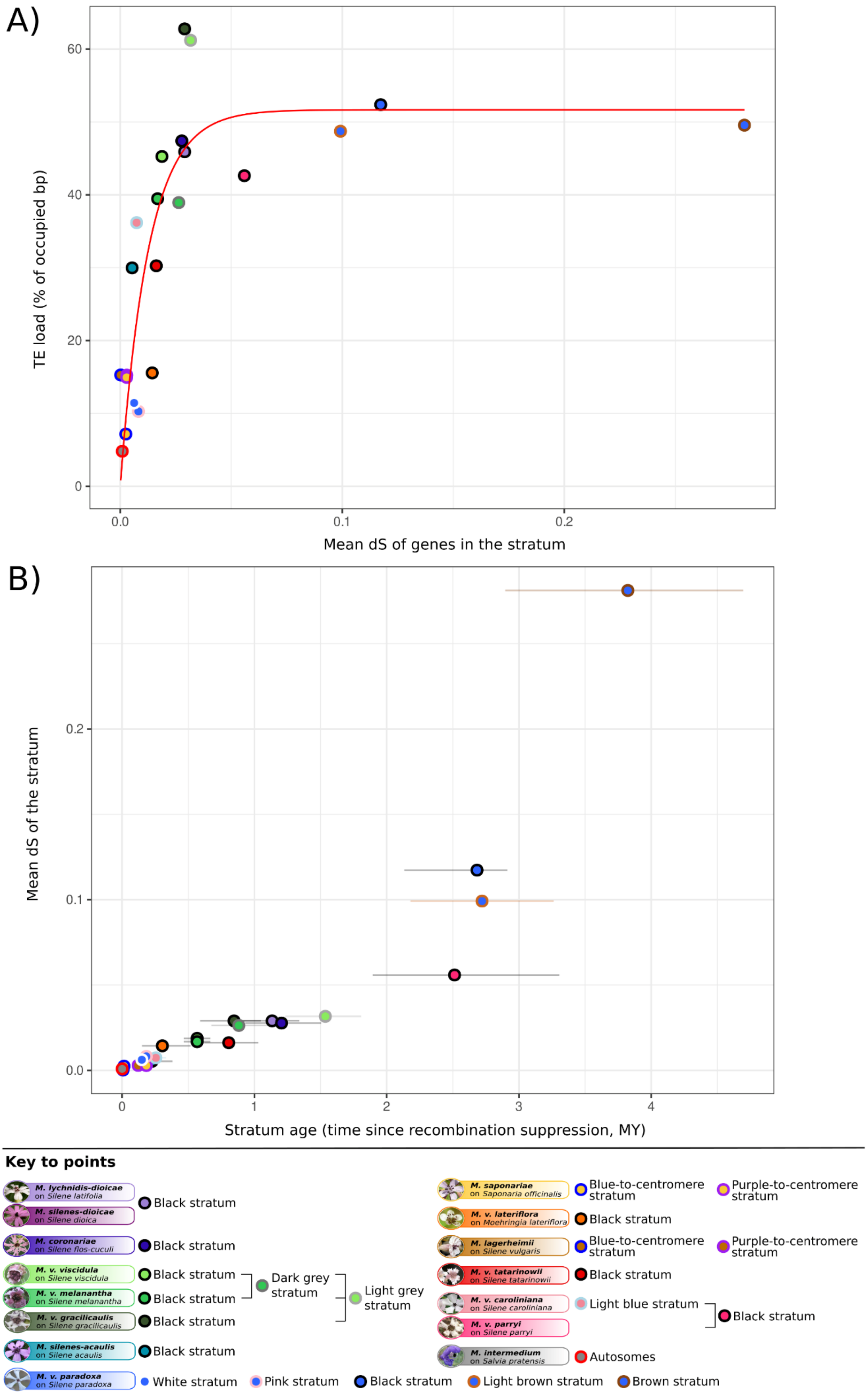
A)
TE load (% of occupied bp)
Mean dS of genes in the stratum
0
20
40
60
0.0
0.1
0.2
B)
Mean dS of the stratum
Stratum age (time since recombination suppression, MY)
0.0
0.1
0.2
0
1
2
3
4
Key to points
M. lychnidis-dioicae on Silene latifolia
M. silenes-dioicae on Silene dioica
Black stratum
M. coronariae on Silene flos-cuculi
Black stratum
M. v. viscidula on Silene viscidula
Black stratum
M. v. melanantha on Silene melanantha
Black stratum
M. v. gracilicaulis on Silene gracilicaulis
Black stratum
Dark grey stratum
Light grey stratum
M. silenes-acaulis on Silene acaulis
Black stratum
M. v. paradoxa on Silene paradoxa
White stratum
Pink stratum
Black stratum
Light brown stratum
Brown stratum
M. saponariae on Saponaria officinalis
Blue-to-centromere stratum
Purple-to-centromere stratum
M. v. lateriflora on Moehringia lateriflora
Black stratum
M. lagerheimii on Silene vulgaris
Blue-to-centromere stratum
Purple-to-centromere stratum
M. v. tatarinowii on Silene tatarinowii
Black stratum
M. v. caroliniana on Silene caroliniana
Light blue stratum
M. v. parryi on Silene parryi
Black stratum
M. intermedium on Salvia pratensis
Autosomes

Supplementary Fig. S19: Relationship between synonymous divergence (d_S), transposable element (TE) load and stratum age in *Microbotryum* mating-type chromosomes. A) Percentage of base pairs occupied by TEs in non-recombining regions (means in a_1 and a_2 genomes and means across species sharing the same strata) as a function of the mean d_S of the genes present in the stratum. The red curve shows the prediction of the best model corresponding to a negative exponential model. **B)** Relationship between the stratum age in million years (MY) and the mean d_S of the genes contained in this stratum. Each dot corresponds to an independent evolutionary stratum in A) and B). The error bars correspond to the 95% confidence interval of the age estimates of the evolutionary strata based on allele genealogies (n = 10,000,000 sampled trees).

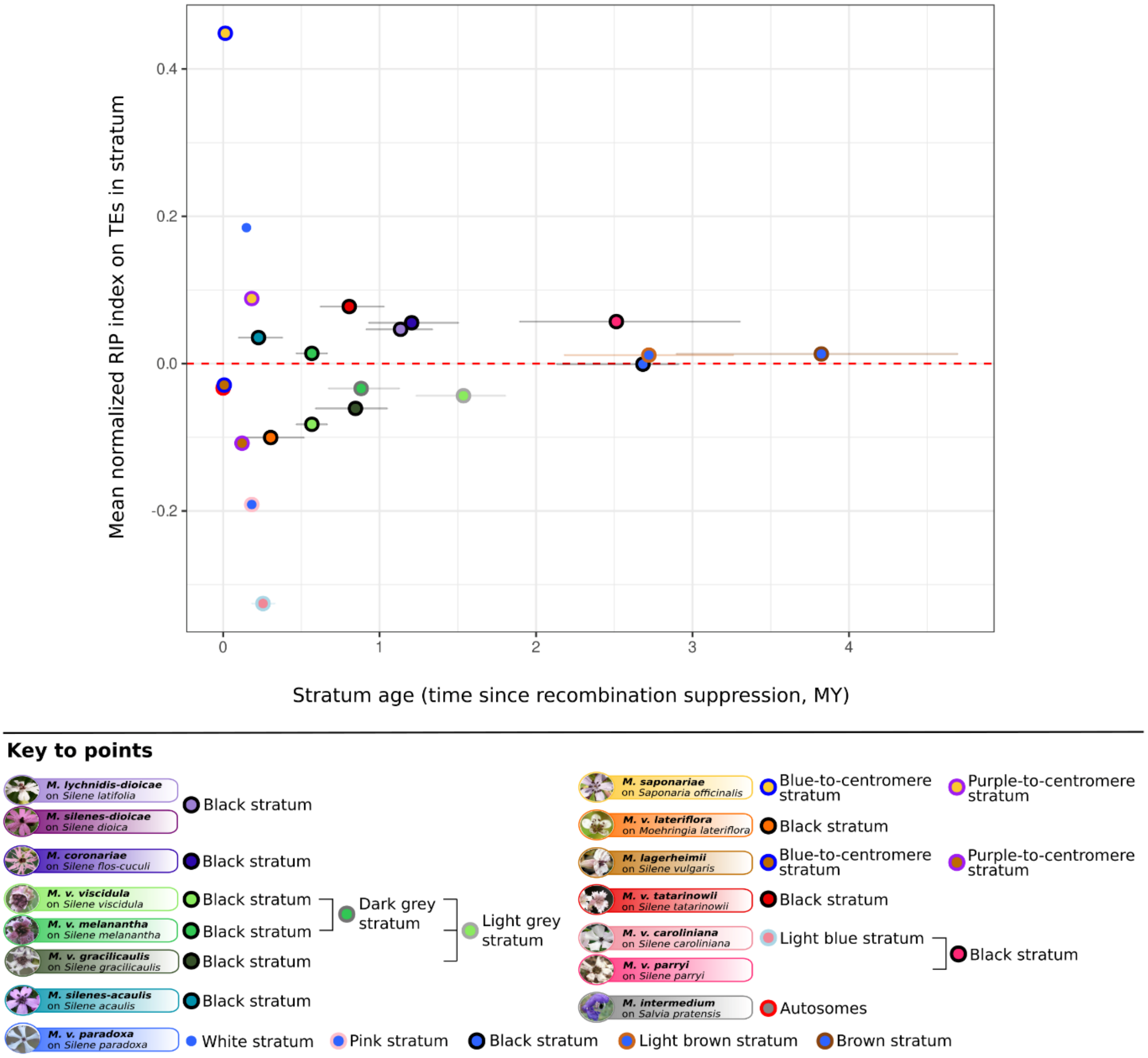

Supplementary Figure 20: Relationship between the abundance of transposable element (TE) inactivation footprints by repeat-induced point mutation (RIP) in the non-recombining regions of the *Microbotryum* mating-type chromosomes and time since recombination suppression in million years (MY). Mean normalized RIP index on TEs in non-recombining regions as a function of their age with confidence intervals. The red dashed line separates the RIP index values suggesting RIP mutations (> 0) or no RIP activity (≤ 0). The two-sided Pearson correlation test was not significant (p-value = 0.9). The error bars correspond

to the 95% confidence interval of the age estimates of the evolutionary strata based on allele genealogies (n = 10,000,000 sampled trees).

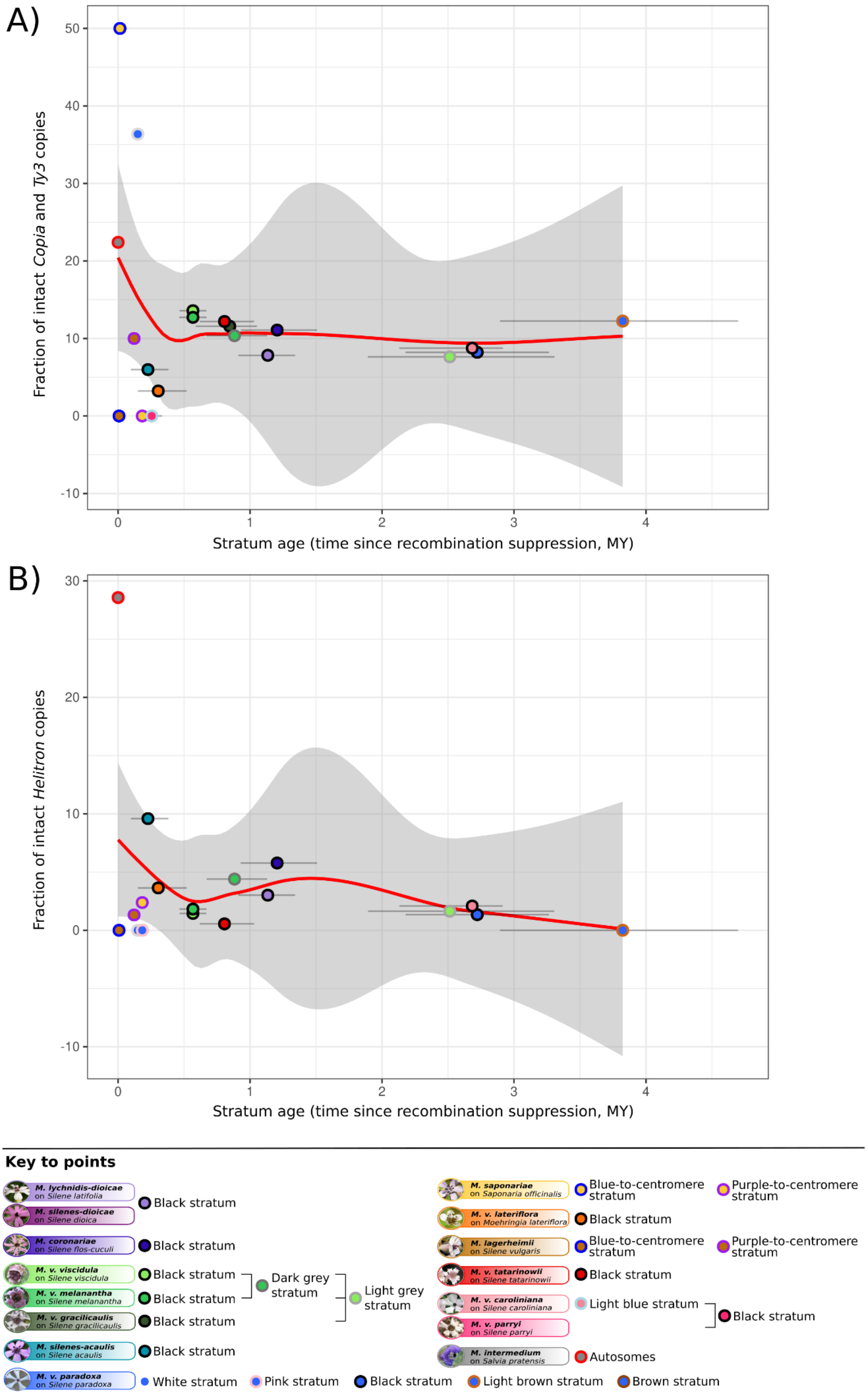
A)
Fraction of intact Copia and Ty3 copies
Stratum age (time since recombination suppression, MY)
B)
Fraction of intact Helitron copies
Stratum age (time since recombination suppression, MY)
Key to points
M. lychnidis-dioicae on Silene latifolia
M. silenes-dioicae on Silene dioica
Black stratum
M. coronariae on Silene flos-cuculi
Black stratum
M. v. viscidula on Silene viscidula
Black stratum
M. v. melanantha on Silene melanantha
Black stratum
Dark grey stratum
Light grey stratum
M. v. gracilicaulis on Silene gracilicaulis
Black stratum
M. silenes-acaulis on Silene acaulis
Black stratum
M. v. paradoxa on Silene paradoxa
White stratum
Pink stratum
Black stratum
Light brown stratum
Brown stratum
M. saponariae on Saponaria officinalis
Blue-to-centromere stratum
Purple-to-centromere stratum
M. v. lateriflora on Moehringia lateriflora
Black stratum
M. lagerheimii on Silene vulgaris
Blue-to-centromere stratum
Purple-to-centromere stratum
M. v. tatarinowii on Silene tatarinowii
Black stratum
M. v. caroliniana on Silene caroliniana
Light blue stratum
M. v. parryi on Silene parryi
Black stratum
M. intermedium on Salvia pratensis
Autosomes

Supplementary Figure 21: Relationship between the fraction of intact *Copia, Ty3* and *Helitron* copies and stratum age in *Microbotryum* mating-type chromosomes. Fraction of intact *Copia* and *Ty3* copies (5-7.5 kb for intact copies) **(A)** and *Helitron* copies (5-11 kb for intact copies) **(B)** in non-recombining regions (means in a_1 and a_2 genomes and means across species sharing the same strata) as a function of the time since recombination suppression in million years (MY). Each dot corresponds to an independent evolutionary stratum in A) and B). The red curve shows the smooth curve obtained by local regression and the grey area shows the 95% confidence intervals of the prediction. The error bars correspond to the 95% confidence interval of the age estimates of the evolutionary strata based on allele genealogies (n = 10,000,000 sampled trees).

Supplementary Tables 1-4.

Supplementary Table 1: Comparisons of transposable element load between genomic compartments in *Microbotryum* genomes				
Dataset: all annotations				
Kolmogorov-Smirnov two-sided test of normality p-value				**0.03657**
ANOVA	df	n	F-value (variation among sample means / variation within groups)	P-value
	**2**	**45**	**19.14**	**0.00000124**
Post-hoc Tukey test	Comparison	Average difference	Confidence interval (0.95%)	Adjusted p-value
	PAR-autosomes	0.01609511	[-0.08532246;0.1175127]	0.9214801
	**NRR-autosomes**	**0.23126343**	**[0.12984586;0.3326810]**	**0.0000054**
	**NRR-PAR**	**0.21516832**	**[0.11375075;0.3165859]**	**0.000018**
Dataset: ClassI:LTR:Copia				
Kolmogorov-Smirnov two-sided test of normality p-value				**0.033**
ANOVA	df	n	F-value (variation among sample means / variation within groups)	P-value
	**2**	**45**	**16.76**	**0.00000446**
Post-hoc Tukey test	Comparison	Average difference	Confidence interval (0.95%)	Adjusted p-value
	PAR-autosomes	-0.4256693	[-3.712044;2.860705]	0.9469469
	**NRR-autosomes**	**6.5592362**	**[3.272862;9.845611]**	**0.0000508**
	**NRR-PAR**	**6.9849055**	**[3.698531;10.271280]**	**0.0000184**
Dataset: ClassI:LTR :Ty3				
Kolmogorov-Smirnov two-sided test of normality p-value				**0.04405**
ANOVA	df	n	F-value (variation among sample means / variation within groups)	P-value
	**2**	**45**	**12.58**	**0.0000522**
Post-hoc Tukey test	Comparison	Average difference	Confidence interval (0.95%)	Adjusted p-value

	PAR-autosomes	-1.598686	[-4.808716;1.611345]	0.4540093
	**NRR-autosomes**	**4.771639**	**[1.561609;7.981670]**	**0.0022802**
	**NRR-PAR**	**6.370325**	**[3.160294;9.580355]**	**0.0000556**
Dataset: ClassII:Helitron				
Kolmogorov-Smirnov two-sided test of normality p-value				0.01576
ANOVA	df	n	F-value (variation among sample means / variation within groups)	P-value
	**2**	**42**	**29.43**	**0.0000000162**
Post-hoc Tukey test	Comparison	Average difference	Confidence interval (0.95%)	Adjusted p-value
	PAR-autosomes	0.4740657	[-1.430914;2.379046]	0.8175409
	**NRR-autosomes**	**5.4155247**	**[3.510545;7.320505]**	**0.0000001**
	**NRR-PAR**	4.941459	[3.036479;6.846439]	0.0000006
Dataset: ClassII:TIR				
Kolmogorov-Smirnov two-sided test of normality p-value				0.3261
ANOVA	df	n	F-value (variation among sample means / variation within groups)	P-value
	**2**	**42**	**6.527**	**0.00347**
Post-hoc Tukey test	Comparison	Average difference	Confidence interval (0.95%)	Adjusted p-value
	PAR-autosomes	0.5401959	[-0.1007642;1.181156]	0.1130969
	**NRR-autosomes**	**0.9508424**	**[0.3098823;1.591802]**	**0.0024011**
	NRR-PAR	0.4106465	[-0.2303136;1.051607]	0.274496

PAR = Pseudo-autosomal region

NRR = Non-recombining region

df = degree of freedom

Significant tests (p<0.01) in **bold**

Supplementary Table 2: Comparisons of the proportion of transposable element (TE) base pairs affected by the repeat-induced point mutation (RIP) between genomic compartments for the amplified TE categories versus other categories in *Microbotryum* genomes					
TE_percentage_RIPed ~ species + TE_category + compartment + TE_category:compartment					
Kolmogorov-Smirnov two-sided test of normality		n	175	p-value	**0.00001255**
ANOVA	factor	df	F-value (variation among sample means / variation within groups)		P-value
	species	14	1.746		0.0522
	**TE_category**	**3**	**13.931**		**0.0000000469**
	**compartment**	**2**	**4.458**		**0.0132**
	TE_category:compartment	6	1.097		0.3671
TE_percentage_RIPed ~ TE_category + compartment					
Kolmogorov-Smirnov two-sided test of normality		n	175	p-value	**0.00001255**
ANOVA	factor	df	F-value (variation among sample means / variation within groups)		P-value
	**TE_category**	**3**	**12.895**		**0.000000126**
	**compartment**	**2**	**4.103**		**0.0182**
Post-hoc Tukey test	Comparison		Average difference	Confidence interval (0.95%)	Adjusted p-value
	ClassI:LTR:Ty3-ClassI:LTR:Copia		-1.09186	[-6.308312;4.124593]	0.9482827
	ClassII:Helitron-ClassI:LTR:Copia		2.103335	[-3.144778;7.351448]	0.7262366
	**others-ClassI:LTR:Copia**		**10.123734**	**[4.966900;15.280568]**	**0.0000055**
	ClassII:Helitron-ClassI:LTR:Ty3		3.195194	[-2.111512;8.5019]	0.4029727
	**others-ClassI:LTR:Ty3**		**11.215593**	**[5.999141;16.432046]**	**0.0000006**
	**others-ClassII:Helitron**		**8.020399**	**[2.772286;13.268512]**	**0.0006202**
	**PAR-NRR**		**-4.154913**	**[-8.294795;-0.01503028]**	**0.0489512**
	**autosomes-NRR**		**-4.45995**	**[-8.563988;-0.35591108]**	**0.0295765**
	autosomes-PAR		-0.305037	[-4.444919;3.83484537]	0.9834066

PAR= Pseudo-autosomal regions; NRR=Non-recombining regions; df = degree of freedom

Significant tests (**p<0.05**) in **bold**

Supplementary Table 3: Comparisons of the age of the *Copia* and *Ty3* copies (number of substitutions between 5’-LTR and 3’-LTR sequences within copy) between the non-recombining regions and the autosomes in *Microbotryum* genomes					
number_substitutions_5-3LTR ~ compartment + species + TE_category + species:TE_category					
Kolmogorov-Smirnov two-sided test of normality		n	1502	p-value	**< 2.2e-16**
ANOVA	factor	df		F-value (variation among sample means / variation within groups)	P-value
	**compartment**	**1**		**6.164**	**0.01315**
	**species**	**9**		**2.426**	**0.00978**
	TE_category	1		1.465	0.22638
	species:TE_category	1		0.026	0.87165
number_substitutions_5-3LTR ~ compartment + TE_category					
Kolmogorov-Smirnov two-sided test of normality		n	1502	p-value	**0.00001255**
ANOVA	factor	df		F-value (variation among sample means / variation within groups)	P-value
	**species**	**9**		**2.427**	**0.00975**
	**compartment**	**1**		**6.166**	**0.01313**
Post-hoc Tukey test	Comparison		Average difference	Confidence interval (0.95%)	Adjusted p-value
	**autosomes-NRR**		**12.72919**	**[2.673617;22.78476]**	**0.0131336**
	MsdSdi-MvSl		4.673821	[-28.556714;37.904357]	0.9999893
	MviLyf-MvSl		27.572742	[-11.675615;66.821098]	0.4392398
	MspSsc-MvSl		7.703014	[-30.788360;46.194387]	0.9997858
	MviSco-MvSl		-14.040164	[-50.912550;22.832223]	0.9714317
	MspSra-MvSl		11.508646	[-25.231817;48.249110]	0.9927029
	MsaSac-MvSl		-17.919403	[-57.913122;22.074315]	0.9212459
	MviSpa-MvSl		9.914503	[-20.484259;40.313264]	0.9902148
	MviSic-MvSl		2.808246	[-43.268053;48.884545]	1
	MspSpr-MvSl		-8.904892	[-50.687846;32.878061]	0.9996401
	MviLyf-MsdSdi		22.89892	[-12.949777;58.747617]	0.5821104
	MspSsc-MsdSdi		3.029192	[-31.989109;38.047493]	0.9999998
	MviSco-MsdSdi		-18.713985	[-51.944520;14.516550]	0.7452268
	MspSra-MsdSdi		6.834825	[-26.249268;39.918918]	0.9997214
	MsaSac-MsdSdi		-22.593225	[-59.256464;14.070014]	0.6326974

	MviSpa-MsdSdi	5.240681	[-20.620105;31.101468]	0.9997624
	MviSic-MsdSdi	-1.865576	[-45.082713;41.351561]	1
	MspSpr-MsdSdi	-13.578714	[-52.185848;25.028420]	0.9833173
	MspSsc-MviLyf	-19.869728	[-60.642837;20.903381]	0.8741047
	**MviSco-MviLyf**	**-41.612905**	**[-80.861262;-2.364549]**	**0.0275385**
	MspSra-MviLyf	-16.064095	[-55.188541;23.060351]	0.9535657
	**MsaSac-MviLyf**	**-45.492145**	**[-87.686435;-3.297855]**	**0.022853**
	MviSpa-MviLyf	-17.658239	[-50.898957;15.582479]	0.8050978
	MviSic-MviLyf	-24.764496	[-72.763308;23.234316]	0.8309966
	MspSpr-MviLyf	-36.477634	[-80.371549;7.416281]	0.2030419
	MviSco-MspSsc	-21.743177	[-60.234551;16.748196]	0.7417881
	MspSra-MspSsc	3.805633	[-34.559385;42.170651]	0.9999995
	MsaSac-MspSsc	-25.622417	[-67.113506;15.868672]	0.6297974
	MviSpa-MspSsc	2.211489	[-30.131944;34.554922]	1
	MviSic-MspSsc	-4.894768	[-52.276603;42.487067]	0.9999993
	MspSpr-MspSsc	-16.607906	[-59.826283;26.610471]	0.9696546
	MspSra-MviSco	25.54881	[-11.191653;62.289273]	0.4548329
	MsaSac-MviSco	-3.87924	[-43.872958;36.114479]	0.9999996
	MviSpa-MviSco	23.954666	[-6.444095;54.353428]	0.2708295
	MviSic-MviSco	16.848409	[-29.227890;62.924708]	0.9782187
	MspSpr-MviSco	5.135271	[-36.647682;46.918224]	0.9999967
	MsaSac-MspSra	-29.42805	[-69.300174;10.444074]	0.3645356
	MviSpa-MspSra	-1.594144	[-31.832752;28.644465]	1
	MviSic-MspSra	-8.700401	[-54.671197;37.270396]	0.9998654
	MspSpr-MspSra	-20.413539	[-62.080119;21.253042]	0.8704866
	MviSpa-MsaSac	27.833906	[-6.283675;61.951487]	0.2255735
	MviSic-MsaSac	20.727649	[-27.882535;69.337833]	0.9413559
	MspSpr-MsaSac	9.014511	[-35.547130;53.576151]	0.9997658
	MviSic-MviSpa	-7.106257	[-48.185901;33.973387]	0.9999369
	MspSpr-MviSpa	-18.819395	[-55.017834;17.379044]	0.8244787
	MspSpr-MviSic	-11.713138	[-61.805729;38.379452]	0.9992357

PAR= Pseudo-autosomal regions; NRR=Non-recombining regions; df = degree of freedom

Significant tests (**p<0.05**) in **bold**

Supplementary Table 4: Selection and summary of models for the tempo of transposable element accumulation in the non-recombining regions				
Kolmogorov-Smirnov two-sided test of normality				
D				**0.1935**
P-value				**0.3378**
Linear model				
AIC				181.7345
Parametric coefficients	Estimate	Standard error	t-value	Pr(>|t|)
**Intercept**	**22.13**	**3.947**	**5.607**	**0.0000173**
**stratum_age**	**11.505**	**2.799**	**4.11**	**0.000544**
Linear model with smoothing splines				
AIC				163.5435
Parametric coefficients	Estimate	Standard error	t-value	Pr(>|t|)
**Intercept**	**32.968**	**1.867**	**17.66**	**0.000000000000877**
**s(stratum_age)**	**3.072**	**3.683**	**15.95**	**0.00000319**
Logarithmic model				
AIC				184.2805
Parametric coefficients	Estimate	Standard error	t-value	Pr(>|t|)
**Intercept**	**37.6266**	**3.3716**	**11.16**	**0.000000000485**
**log(stratum_age)**	**2.9569**	**0.8245**	**3.586**	**0.00185**
Logarithmic model with smoothing splines				
AIC				165.2281
Parametric coefficients	Estimate	Standard error	t-value	Pr(>|t|)
**Intercept**	**32.968**	**1.908**	**17.28**	**0.00000000000343**
**s(log(stratum_age))**	**4.065**	**4.75**	**14.49**	**0.0000175**
Linear model with ancestral size as covariable				
AIC				181.8446
Parametric coefficients	Estimate	Standard error	t-value	Pr(>|t|)
**Intercept**	**24.04**	**4.143**	**5.801**	**0.0000137**
**stratum_age**	**10.81**	**2.802**	**3.856**	**0.00106**
ancestral_stratum_size	-0.0000009222	0.0000007049	-1.308	0.20641
Linear model with smoothing splines with ancestral size as covariable				

AIC				164.9114
Parametric coefficients	Estimate	Standard error	t-value	Pr(>|t|)
**Intercept**	**33.533**	**2.011**	**16.669**	**0.0000000000046**
ancestral_stratum_size	-0.0000004127	0.0000004811	-0.858	0.403
**s(stratum_age)**	**2.774**	**3.348**	**17.19**	**0.0000166**
Logarithmic model with ancestral size as covariable				
AIC				172.5754
Parametric coefficients	Estimate	Standard error	t-value	Pr(>|t|)
**Intercept**	**38.878**	**2.549**	**15.212**	**0.0000000000043**
**log(stratum_age)**	**8.237**	**1.443**	**5.709**	**0.0000167**
**ancestral_stratum_size**	**0.000005291**	**0.000001306**	**4.053**	**0.000679**
Logarithmic model with smoothing splines with ancestral size as covariable				
AIC				168.9572
Parametric coefficients	Estimate	Standard error	t-value	Pr(>|t|)
**Intercept**	**29.28**	**3.961**	**7.392**	**0.000000784**
ancestral_stratum_size	0.000002718	0.000002491	1.091	0.29
**s(log(stratum_age))**	**2.175**	**2.567**	**17.58**	**0.0000315**
Negative exponential model				
AIC				**161.4592**
Parametric coefficients	Estimate	Standard error	t-value	Pr(>|t|)
**a:(Intercept)**	**50.55787**	**3.43574**	**14.7153**	**0.000000000003425**
**c:(Intercept)**	**2.58939**	**0.55653**	**4.6527**	**0.0001534**
Negative exponential model with phylogenetic tree				
Parametric coefficients	Estimate	Standard error	t-value	Pr(>|t|)
Intercept	3.95998	3.70399	1.0691	0.2978
**stratum_age.negExp**	**0.90213**	**0.10206**	**8.8389**	**2.416e-08**
Branch length transformation	lower bound (0)	upper bound (1)	95% confidence interval	
lambda [ML]	1	0.0010937	[NA;0,787]	

Significant parameters (**p<0.01**) in **bold**

Lowest AIC score and significant parameters in **bold**

Supplementary Note 1: Datasets published on Figshare.

- **Gene prediction in *Microbotryum* species genomes**: gff3 files of the gene predicted in the haploid genomes of 16 *Microbotryum* species and a red yeast (*Rhodothorula babjavae*), for each mating type, using EuGene 4.2a (Froissac *et al.,* 2008).
https://doi.org/10.6084/m9.figshare.20600775.v1

- **Transposable element detection and annotation in *Microbotryum* species genomes:** gff3 files of transposable element sequences detected in the haploid genomes of 16 *Microbotryum* species as well as a red yeast (*Rhodothorula babjavae*), for each mating type.
https://doi.org/10.6084/m9.figshare.20600721.v1

- **Table matching short ids and file names to species name and mating type:** Matching table for the other dataset in this project. https://doi.org/10.6084/m9.figshare.20600664.v1

- **Telomere coordinates in *Microbotryum* species genomes:** Telomere identification in the haploid genomes of 16 *Microbotryum* species, for each mating type.
https://doi.org/10.6084/m9.figshare.20600544.v1

- **Centromere prediction in *Microbotryum* species genomes:** Centromere coordinates of haploid genomes of 16 *Microbotryum* species, for each mating type.
https://doi.org/10.6084/m9.figshare.20599782.v1
