# Supplementary material for: Dynamics of transposable element accumulation in the non-recombining regions of mating-type chromosomes in anther-smut fungi
Source: Nat Commun. 2023 Sep 14;14:5692. doi: 10.1038/s41467-023-41413-4 (PMC10502011; doi:10.1038/s41467-023-41413-4)
Supplement: Supplementary file 3 — Reporting Summary [file 41467_2023_41413_MOESM3_ESM.pdf]

nature portfolio

Corresponding author(s): Marine Duhamel

Last updated by author(s): 17/08/2023

# Reporting Summary

Nature Portfolio wishes to improve the reproducibility of the work that we publish. This form provides structure for consistency and transparency in reporting. For further information on Nature Portfolio policies, see our Editorial Policies and the Editorial Policy Checklist.

## Statistics

For all statistical analyses, confirm that the following items are present in the figure legend, table legend, main text, or Methods section.

| n/a | Confirmed | |
|---|---|---|
| ☐ | ☒ | The exact sample size (*n*) for each experimental group/condition, given as a discrete number and unit of measurement |
| ☐ | ☒ | A statement on whether measurements were taken from distinct samples or whether the same sample was measured repeatedly |
| ☐ | ☒ | The statistical test(s) used AND whether they are one- or two-sided<br>*Only common tests should be described solely by name; describe more complex techniques in the Methods section.* |
| ☐ | ☒ | A description of all covariates tested |
| ☐ | ☒ | A description of any assumptions or corrections, such as tests of normality and adjustment for multiple comparisons |
| ☐ | ☒ | A full description of the statistical parameters including central tendency (e.g. means) or other basic estimates (e.g. regression coefficient) AND variation (e.g. standard deviation) or associated estimates of uncertainty (e.g. confidence intervals) |
| ☐ | ☒ | For null hypothesis testing, the test statistic (e.g. *F*, *t*, *r*) with confidence intervals, effect sizes, degrees of freedom and *P* value noted<br>*Give P values as exact values whenever suitable.* |
| ☒ | ☐ | For Bayesian analysis, information on the choice of priors and Markov chain Monte Carlo settings |
| ☒ | ☐ | For hierarchical and complex designs, identification of the appropriate level for tests and full reporting of outcomes |
| ☐ | ☒ | Estimates of effect sizes (e.g. Cohen's *d*, Pearson's *r*), indicating how they were calculated |

*Our web collection on statistics for biologists contains articles on many of the points above.*

## Software and code

Policy information about availability of computer code

Data collection

No software was used for data collection

Data analysis

The transposable element (TE) detection pipeline is available at https://gitlab.com/marine.c.duhamel/microtep. This pipeline combined long-terminal (LTR) retrotransposon detected by LTRharvest from GenomeTools 1.5.10 and TE consensus sequence detection by RepeatModeler 1.0.11, and enriched the TE detection by BLASTN (2.6.0+). TE sequence annotation was performed by BLASTN and BLASTX (2.6.0+) as well as pfam_scan.pl (ftp://ftp.ebi.ac.uk/pub/databases/Pfam/Tools/). We built the TE genealogies based on the LTR sequences detected by LTRHarvest from GenomeTools 1.5.10 and oriented using RepeatProteinMask version 1.0.11. We aligned the LTR sequences using MAFFT v7.310 and built the genealogies using IQ-TREE 2.0.4. We drew the trees using the R packages phytools 1.0-3 and ggtree 3.4.0, and extracted the sequence pairwise divergence using ape 5.6-2 R packages. We estimated the Kimura divergence between two LTR sequences using MEGA11.
After removing the genes overlapping with annotated TEs, we built orthologous groups using Markov clustering (mcl 14-137) of high-scoring pairs parsed with orthAgogue based on all-vs-all BLASTP 2.6.0+ on protein sequences. We independently aligned the coding sequences of 3,669 single-copy genes present in all Microbotryum species and the outgroup Rhodothorula babjavae using MUSCLE as implemented in TranslatorX v1.1. We used IQTREE 2.0.4 to build the maximum likelihood species tree with the GTR+GAMMA model of substitution chosen according to the Akaike information criterion by Model Finder implemented in IQ-TREE 2.0.4. We assessed the robustness of the nodes with 1000 ultrafast bootstraps and SH-like approximate likelihood ratio tests (SH-aLRT) from the concatenated alignment, implemented in IQ-TREE 2.0.4.
We searched for potential horizontal TE transfers by blast (BLASTN 2.6.0+).
We identified the centromeres based on a conserved centromeric motif previously identified and blasted (BLAST 2.6.0+) against the genome. The script used to compute the RIP index is available at https://gitlab.com/marine.c.duhamel/ripmic. We plotted TE and gene density, as well as the RIP index along the contigs using the evobiR 1.1 and ggplot2 3.4.0 R packages.

We visualized the rearranged evolutionary strata on the mating-type contigs using circos 0.69-6.
We calculated the synonymous divergence (dS) from the alignment of a1 and a2 allele sequences using MUSCLE implemented in TranslatorX v1.1. We computed the dS and its standard error using the yn00 v4.9f program of the PAML package and plotted them using the ggplot2 2 3.4.0 in R.
We built non-linear regression models using the drc 3.2-0 R package, local regressions using the fANCOVA 0.6-1 R package and tested the presence of phylogenetic signal using the caper package version 1.0.1.
We performed all R analyses in R version 4.2.1.

For manuscripts utilizing custom algorithms or software that are central to the research but not yet described in published literature, software must be made available to editors and reviewers. We strongly encourage code deposition in a community repository (e.g. GitHub). See the Nature Portfolio guidelines for submitting code & software for further information.

## Data

Policy information about availability of data

All manuscripts must include a data availability statement. This statement should provide the following information, where applicable:

- Accession codes, unique identifiers, or web links for publicly available datasets
- A description of any restrictions on data availability
- For clinical datasets or third party data, please ensure that the statement adheres to our policy

The datasets supporting the conclusions of this article are included within the article (Supplementary Tables 1 - 4 and Supplementary Data 1 - 6). All data generated or analysed during this study are included in this published article and the supplementary information files. Source data are provided with this paper. rRNA or overlapping Microbotryum ribosomal sequences were downloaded from https://www.arb-silva.de/ and http://combio.pl/rrna/taxId/5272/ and mitochondrial DNA sequences from GenBank (NC_020353). TE sequence similarity searches were performed using Repbase database 23.05. Sequencing data and genome assemblies were published previously and available at GenBank under the BioProjects PRJEB12080, PRJNA437556, PRJEB16741, PRJEB15277, PRJNA771266 and PRJNA437556. All fungal strains are available upon request.

## Research involving human participants, their data, or biological material

Policy information about studies with human participants or human data. See also policy information about sex, gender (identity/presentation), and sexual orientation and race, ethnicity and racism.

| | |
|---|---|
| Reporting on sex and gender | not applicable |
| Reporting on race, ethnicity, or other socially relevant groupings | not applicable |
| Population characteristics | not applicable |
| Recruitment | not applicable |
| Ethics oversight | not applicable |

Note that full information on the approval of the study protocol must also be provided in the manuscript.

# Field-specific reporting

Please select the one below that is the best fit for your research. If you are not sure, read the appropriate sections before making your selection.

☐ Life sciences ☐ Behavioural & social sciences ☒ Ecological, evolutionary & environmental sciences

For a reference copy of the document with all sections, see nature.com/documents/nr-reporting-summary-flat.pdf

# Ecological, evolutionary & environmental sciences study design

All studies must disclose on these points even when the disclosure is negative.

| | |
|---|---|
| Study description | In this study, we investigated the temporal dynamics of transposable element (TE) accumulation in the non-recombining regions of |
| Research sample | We used the assemblies of a1 and a2 haploid genome of 16 Microbotryum species (Microbotryum lychnidis-dioicae 1064, M. We used the assemblies of a1 and a2 haploid genome of 16 Microbotryum species (Microbotryum lychnidis-dioicae 1064, M. silenesdioicae<br>1303, M. coronariae 1247, M. violaceum viscidula 01506, M. v. melanantha 1296, M. v. gracilicaulis 01299, M. silenes-acaulis 1248, M. v. paradoxa 1252, M. lagerheimii 1253, M. v. lateriflora 01509, M. saponariae 1268 and 1269 respectively, M. v. tatarinowii 1400, M. v. caroliniana 1250, M. v. parryi 01510, M. scabiosae 1118 and M. intermedium 01389) and the red yeast Rhodothorula babjavae (PTZ001 and 7809, respectively) previously published and available on the NCBI database. We chose this dataset because the genome assemblies were already available and of good quality for TE detection, and because evolutionary strata had already been described, delimitated and dated. |

| | |
|---|---|
| Sampling strategy | The assemblies of a1 and a2 haploid genome of 16 Microbotryum species represented 21 evolutionary strata of different ages and one almost fully recombining genome used as a one day-old evolutionary stratum and were already available. |
| Data collection | We used all high-quality assemblies of Microbotryum genomes available in the literature (Branco et al., 2017; 2018; Carpentier et al., 2019; 2022; Duhamel et al., 2022) and downloaded from NCBI. |
| Timing and spatial scale | We used all high-quality assemblies of Microbotryum genomes available in the literature regarless of time of collection or spatial scale |
| Data exclusions | We used all high-quality assemblies of Microbotryum genomes available in the literature and excluded no available genome. |
| Reproducibility | We give above all pipelines, databases and genome accessions for reproducing the analyses. |
| Randomization | We analysed genomic data and did not performed any experiment, so there was no need or way to randomization. |
| Blinding | We analysed genomic data and did not performed any experiment, so there was no need or way to blinding. |

Did the study involve field work? ☐ Yes ☒ No

# Reporting for specific materials, systems and methods

We require information from authors about some types of materials, experimental systems and methods used in many studies. Here, indicate whether each material, system or method listed is relevant to your study. If you are not sure if a list item applies to your research, read the appropriate section before selecting a response.

## Materials & experimental systems

| n/a | Involved in the study |
|---|---|
| ☒ | ☐ Antibodies |
| ☒ | ☐ Eukaryotic cell lines |
| ☒ | ☐ Palaeontology and archaeology |
| ☒ | ☐ Animals and other organisms |
| ☒ | ☐ Clinical data |
| ☒ | ☐ Dual use research of concern |
| ☒ | ☐ Plants |

## Methods

| n/a | Involved in the study |
|---|---|
| ☒ | ☐ ChIP-seq |
| ☒ | ☐ Flow cytometry |
| ☒ | ☐ MRI-based neuroimaging |
