# Supplementary material for: Dynamics of transposable element accumulation in the non-recombining regions of mating-type chromosomes in anther-smut fungi
Source: Nat Commun. 2023 Sep 14;14:5692. doi: 10.1038/s41467-023-41413-4 (PMC10502011; doi:10.1038/s41467-023-41413-4)
Supplement: Supplementary file 4 — Description of additional supplementary files [file 41467_2023_41413_MOESM4_ESM.pdf]

Description of Additional Supplementary Files

Supplementary Data 1

Description: Transposable element (TE) content, age and ancestral size of the strata in Microbotryum species

Supplementary Data 2

Description: Genomic percentage of each transposable element annotation in Microbotryum species and Rhodothorula babjavae

Supplementary Data 3

Description: BLAST hits results of Microbotryum Copia and Ty3 retrotransposons against the Repbase 23.05 database sequences of Arabidopsis thaliana and Dicotyledones

Supplementary Data 4

Description: Genomic percentage transposable element annotation in the different genomic compartments of Microbotryum species and Rhodothorula babjavae

Supplementary Data 5

Description: Coordinates of the centromeres of all Microbotryum genomes of this study

Supplementary Data 6

Description: Coordinates of the telomeres of all Microbotryum genomes of this study
